# Isolating the impact of specific gambling activities and modes on problem gambling and psychological distress in internet gamblers

**DOI:** 10.1186/s12889-019-7738-5

**Published:** 2019-10-25

**Authors:** Sally M. Gainsbury, Douglas J. Angus, Alex Blaszczynski

**Affiliations:** University of Sydney, Science, Brain and Mind Centre, School of Psychology, Camperdown, NSW Australia

**Keywords:** Disordered gambling, Psychological distress, Problem gambling, Internet gambling, Participation frequency, Electronic gaming machines, Addiction, Sports betting, Casino, Harm

## Abstract

**Background:**

Gambling disorder is related to high overall gambling engagement; however specific activities and modalities are thought to have stronger relationships with gambling problems. This study aimed to isolate the relationship between specific gambling activities and modalities (Internet and venue/land-based) to gambling disorder and general psychological distress. Past-month Internet gamblers were the focus of this investigation because this modality may be associated with gambling disorders in a unique way that needs to be separated from overall gambling intensity.

**Methods:**

Australians who had gambled online in the prior 30 days (*N* = 998, 57% male) were recruited through a market research company to complete an online survey measuring self-reported gambling participation, problem gambling severity, and psychological distress.

**Results:**

When controlling for overall gambling frequency, problem gambling was significantly positively associated with the frequency of online and venue-based gambling using electronic gaming machines (EGMs) and venue-based sports betting. Psychological distress was uniquely associated with higher frequency of venue gambling using EGMs, sports betting, and casino card/table games.

**Conclusions:**

This study advances our understanding of how specific gambling activities are associated with disordered gambling and psychological distress in users of Internet gambling services. Our results suggest that among Internet gamblers, online and land-based EGMs are strongly associated with gambling disorder severity. High overall gambling engagement is an important predictor of gambling-related harms, nonetheless, venue-based EGMs, sports betting and casinos warrant specific attention to address gambling-related harms and psychological distress among gamblers.

## Background

Disordered and problem gambling represent important public health concerns and psychopathologies. The prevalence of gambling disorder is estimated at around 1% in various international jurisdictions [1–6]; however there is a significant impact of sub-clinical gambling problems experienced by a broader proportion of the population. These individuals are at-risk of developing more severe gambling problems, in addition to other mental health disorders [7, 8]. Gambling activities are diverse with markable differences between activities in terms of the mechanics, structural characteristics, and environment. For example, the same activity provided in venues or in online modalities may have unique characteristics that can lead to harms. The present study aims to isolate the unique relationship of specific gambling activities and modalities to problem gambling and psychological distress among Internet gamblers. This increased understanding of gambling disorder and psychological distress is essential in guiding treatment and prevention initiatives. This research will enable regulators and other stakeholders to optimise their efforts to counter gambling problems.

### Internet gambling

Internet gambling (also referred to as online, interactive, or remote gambling, incorporating multiple Internet platforms and mobile devices) is no longer a newly emerging phenomenon, but a relatively well-established mode of accessing gambling globally. The legality of Internet gambling differs between jurisdictions with legislative variations ranging from prohibition or partial legalisation, to broad legal access [9, 10]. Many governments include considerations of harms related to Internet gambling in their legislative efforts. Nonetheless, research on the use of Internet gambling and its unique contribution to gambling-related problems is limited.

Initial prevalence studies that included Internet gambling suggested that the rates of gambling problems are significantly higher in populations of online compared to land-based gamblers [2, 11–15]. However, when controlling for involvement in terms of frequency of participation, expenditure, and number of forms used (including land-based), Internet gambling participation does not uniquely predict gambling problems [2, 3, 16–19]. This is consistent with population prevalence studies which have not shown an increase in problem gambling prevalence, despite increases in Internet gambling participation [1, 2, 6]. For example, an analysis across 30 European jurisdictions did not identify any association between prohibitions against online gambling, gambling licensing systems, the extent of legal gambling opportunities and the prevalence of gambling disorder [5].

### Gambling activities and gambling modality

Internet gambling does not represent a specific type of gambling activity, but rather a mode of access. Nonetheless, gambling activities have different features depending on whether they are accessed via Internet-connected devices or in venues, and different propensities and pathways that may contribute to the development of gambling disorders and problems (e.g., [20–26]). For example, venue-based gambling typically uses cash as compared to the credit cards and electronic funds transfers used in Internet gambling, which have been associated with greater expenditure [27, 28]. Social interactions may be limited to those also engaging in online gambling, rather than people who may decide to cease gambling and engage in other activities. That is, although the mechanics are typically similar within gambling activities, the structural characteristics can be markedly different within the same activity in land-based as compared to Internet modalities.

Isolating the impact of specific modes of gambling is critical as many problem gamblers engage in multiple gambling activities and focusing only on overall participation can lead to misleading interpretations. For example, in an Australian national telephone survey, the number of gambling activities used was predictive of increased gambling problem severity. When asked which mode of gambling made the greatest contribution to problems, 58% of those who had gambled online indicated land-based modes; 53.5% indicated that their problems had developed before they first gambled online [2].

It is important to consider a potential interaction between the mode of gambling (i.e., Internet vs. land-based) and specific gambling activities in relation to gambling problems. In an Australian prevalence study, 66.9% of Internet gamblers experiencing problems reported using sports betting, while only 23.1% of land-based gamblers experiencing problems reported using sports betting [2]. Further, problem Internet gamblers were more likely to self-nominate sports or horse betting as causing their problems, in comparison to land-based problem gamblers who indicated EGMs as a causal activity [29]. Very few studies have examined the differences between gambling activities by modality in terms of their contribution to problems.

The interpretation of previous findings is further complicated by the observation that many users of Internet gambling activities also gamble in venues. That is, someone who is considered an “Internet gambler” may not exclusively – or even preferentially – use online modes of access. Given the potential for complementary or compounding patterns of gambling behaviour (e.g., wagering online and in venues) [30, 31], it is necessary to examine the relationship between problem gambling, gambling activities, and gambling modalities.

Due to finite resources, policy makers typically focus efforts to minimise gambling harms on specific activities. For example, electronic gaming machines (EGMs) are often highlighted as a specifically harmful gambling activity. These are often the most commonly reported form of gambling by individuals seeking help, and its participation associated with a greater likelihood of experiencing gambling problems [32–35]. It has been theorised and there is some research to support that features of EGMs may increase harm, including the rapid rate at which bets can be placed and results revealed, the variable reinforcement schedule, the ability to place large bets across multiple lines, and the audio and visual stimulation [33, 36, 37]. An analysis of 18 national prevalence studies indicated that EGMs, casino gambling, illegal gambling, and Internet gambling were consistently most strongly associated with gambling problems. Sports and horse race betting, and bingo were consistently moderately associated, while lottery type activities were consistently weakly associated [38, 39]. However, several recent studies have reported that overall gambling involvement is the most important factor in determining the risk of gambling problems, and that specific activities are not related to problems if overall involvement and intensity are statistically controlled for [3, 19, 40, 41]. These findings do not suggest that all forms of gambling are equally related to problems, but that involvement in multiple compared to single forms is a stronger predictor of gambling problems.

Despite the above findings, many studies have used methodologies that make it difficult to isolate relevant factors including frequency of participation in each form and the mode of gambling access. First, several studies have measured Internet gambling as a discrete gambling activity, rather than a mode of accessing specific gambling activities (e.g., [15, 42], making it impossible to identify the impact of specific types of Internet gambling on problems. Second, studies have statistically controlled for involvement in multiple forms by using the sum of activities gambled on, while retaining the original or transformed activity measures in a regression model. This may produce biased results because of collinearity between the composite measure of involvement and the activity measures it is directly derived from [43]. This method of controlling for involvement also inherently controls for participation in individual activities, distorting and potentially supressing estimates of their impact on problem gambling.

Problem gambling severity is an important factor to consider in establishing the impact of specific activities; however, overall psychological distress is also a critical consideration. Several studies have found that poor mental health and psychological distress are predictive of greater problem gambling severity [2, 44]. One Australian study found that land-based problem gamblers reported greater psychological distress than Internet problem gamblers [29], suggesting that there may be covariates related to distress in addition to the experience of problems. Although gambling disorder is highly comorbid with other mental health disorders [4, 45, 46], most studies do not observe a direction of causality. Therefore, it is important to consider the unique relationship between psychological distress and participation in specific gambling activities, and specific modes of access.

### The present study

This study aimed to investigate the relation of gambling frequency to problem gambling severity and psychological distress to understand the unique contribution of specific gambling activities to these mental health issues. Based on previous literature, we hypothesised that the frequency of involvement in a range of online and land-based gambling activities would be positively correlated with both problem gambling severity and psychological distress. Given the existing literature suggesting that EGM use is related to gambling problems, a secondary hypothesis was that engagement in land-based and online EGMs would be positively related to problem gambling severity. We conducted multiple regressions exploring the unique relationship between participation frequency of each gambling activity by its modality (online and land-based) and 1) problem gambling severity, and 2) psychological distress, as well as investigating any demographic predictors.

## Methods

### Participants

Participants were 1001 Australian Internet users, aged 18–85, self-reporting participation in online gambling at least once in the 30 days prior to completing the survey. The Australian gambling context includes partial legalization and prohibition; sports, esports, and race wagering is provided online through licensed domestic providers with all other forms of gambling prohibited online, however these are available through offshore providers [47]. Land-based venues are highly accessible and legally provide lottery products, EGMs, sports betting, race wagering, poker, and casino card/table games.

Potential participants were required to be age 18 years or older, be active Internet users, and have English comprehension. Participants were recruited from an existing database of potential research participants held by market research company Qualtrics. Overall panel and study response rates were not provided to the research team. The survey was completed between March 30 and April 5, 2017. After removal of participants completing the online survey twice, 998 (99.7%) participants were retained for further analysis (Table 1). All participants provided informed electronic consent. Ethics approval for the study was provided by the University of Sydney Human Research Ethics Committee.

**Table 1:** Demographic characteristics of 998 past month internet gamblers

Demographic Factor	*n = 998*
PGSI Sum [Median, 25th–75th percentile]	1.00 [0.00, 6.00]
Kessler 6 Sum [Median, 25th–75th percentile]	3.00 [0.00, 10.00]
Age (Mean, SD)	48.18 (15.81)
Gender (n, %)	
Male	570 (57.11)
Female	427 (42.79)
Other	1 (0.10)
Relationship Status (n, %)	
Not in a romantic relationship	302 (30.26)
Casually dating (i.e., not exclusive)	39 (3.91)
Exclusively dating	44 (4.41)
Engaged	14 (1.40)
Living together	67 (6.71)
Married or defacto	532 (53.31)
Education (n, %)	
Postgraduate qualification	106 (10.62)
University or college degree	260 (26.05)
Trade/technical certificate/diploma	298 (29.86)
Year 12 or equivalent	201 (20.14)
Year 10 or equivalent	116 (11.62)
Less than Year 10	17 (1.70)
Work Status (n, %)	
Work full-time	404 (40.48)
Work part-time or casual	189 (18.94)
Unemployed	53 (5.31)
Full-time student	37 (3.71)
Full-time home duties	61 (6.11)
Retired	186 (18.64)
Sick or disability pension	51 (5.11)
Other	17 (1.70)
Household Income (n, %)	
Less than $25,000 per year	82 (8.22)
$25,000–$49,999	225 (22.55)
$50,000–$74,999	173 (17.33)
$75,000–$99,999	156 (15.63)
$100,000–$124,999	109 (10.92)
$125,000–$149,999	79 (7.92)
$150,000–$174,999	33 (3.31)
$175,000–$199,999	22 (2.20)
$200,000 or more per year	27 (2.71)
I prefer not to say	92 (9.22)
Born in Australia (n, %)	
Yes	797 (79.86)
No	201 (20.14)
Language Other Than English at Home (n, %)	
Yes	88 (8.82)
No	910 (91.18)
Ethnicity (n, %)	
European	815 (81.66)
East and Southeast Asian	70 (7.01)
South Asian	47 (4.71)
Middle Eastern	19 (1.90)
African	3 (0.30)
Latin, Central and South American	5 (0.50)
Pacific Islander	7 (0.70)
Indigenous Australian	23 (2.30)
Other	9 (0.90)

### Measures

The full survey included items for standard demographic details (e.g., age, gender, household income), online and land-based gambling behaviours, attitudes towards online gambling sites, motivations for engaging in online gambling, problem gambling severity, and psychological harms. Previous papers from this dataset have focused on the use of eSports as a newly introduced form of gambling in Australia [48, 49]. The present exploratory analyses made use of demographic measures, measures of online and venue gambling activity frequency, problem gambling severity, and psychological distress. Analyses were limited to these variables because other survey items (e.g., perceived advantages and disadvantages of gambling sites with or without an Australian license) were designed to answer a different set of research questions than those addressed in the present manuscript.

#### Online gambling frequency

Participants were asked to indicate how often in the preceding 4 weeks they had gambled online for real money on each of the following activity types: lottery-type, EGMs, sports betting, eSports betting, race wagering, poker, casino card/table games, and other. For each activity type, participants were able to indicate if they have gambled on that activity: “not in the past four weeks” (1), “at least once in the past 4 weeks” (2), “at least once per week” (3), or “at least once per day” (4). An ordinal coding scheme was used for all online gambling activity frequency variables.

#### Venue gambling frequency

Participants were asked to indicate how often in the preceding 4 weeks they had gambled in venues for real money on the following activity types: lottery-type, EGMs, sports betting, eSports betting, race wagering, poker, casino card/table games, and other. Response were made using the same format as for the online gambling frequency described above.

#### Problem gambling severity index

The Problem Gambling Severity Index (PGSI) was used to measure problem gambling severity [50]. In the present study, we used the sum score as a count measure of problem gambling severity [2, 16, 51], rather than using the classification categories used in other studies. Several factors motivated our choice to treat the PGSI as a count variable. First, there is considerable debate regarding how low-risk and high-risk categories of the PGSI should be interpreted or scored [52, 53]. Second, the relationship between dependent variables and each level of the canonical PGSI were observed to be non-linear and violated a critical assumption of ordinal logistic regression models. Third, while a binary logistic regression could be applied to these data, the dichotomization of variables had received considerable criticism, and may produce biased results [54, 55]. The internal reliability of the PGSI was extremely good (*α* = 0.95).

To aid in comparison with other studies, we also calculated the proportion of participants classified into each PGSI group. 39.58% (*n* = 395) of the sample were classified as non-problem gamblers, 21.74% (*n* = 217) as low-risk gamblers, 16.73% (*n* = 167) as moderate-risk gamblers, and 21.94% (*n* = 219) as problem gamblers. We note that it is similar to the rates reported by other studies using online panels [56].

#### Kessler psychological distress scale

The Kessler 6 (K6) is a six item self-report scale intended to measure the level of non-specific psychological distress experienced in the preceding 4 weeks, and covers symptoms such as nervousness, feelings of worthlessness, hopelessness, and depression [57]. In addition to its brevity, the K6 has excellent internal reliability, and has been correlated with independent assessments of mental illness and psychological distress [58]. For each item, the following response options were available: “none of the time” (0), “a little of the time” (1), “some of the time” (2), “most of the time” (3) and “all of the time” (4). As with the PGSI, we calculated the sum of responses to each K6 item. The sum K6 score was then used as a count measure of psychological distress. The internal reliability of the K6 was extremely good (***α*** = 0.95).

#### Breadth of online and venue involvement

In keeping with past studies [41], we also calculated breadth variables for online and venue activities. Each of these composite count variables used the total number of activities that participants had engaged in at least once in the preceding 30 days. The total number of online activities could range from one to seven, and the total number of venue activities could range from zero to seven.

### Statistical analyses

Data were analysed using *R* [59]. Relationships between the frequencies of participation in each activity were examined using Spearman’s Rho. Relationships between activity frequency, PGSI scores, and K6 scores were also examined using Spearman’s Rho. To facilitate interpretation of these correlations we report the median and 25th–75th percentiles of PGSI and K6 for each level of activity frequency. This reporting approach was used because of the non-normal distribution of PGSI and K6 scores, and the ordinal nature of the activity frequency variables. The Bonferroni method was used to correct for multiple comparisons when conducting these analyses. The unique contribution of each online or venue gambling activity and potentially related demographic details to PGSI and K6 scores were examined using Quasi-Poisson regressions. We used Quasi-Poisson regressions because of the extremely positively skewed and leptokurtic distributions of the PGSI and K6, and initial examinations which indicated that these variables were over-dispersed (e.g., their variance was greater than their mean). We used a recently developed variance-based method of calculating *R*^*2*^ to derive estimates of the variance accounted for by each regression [60]. These *R*^*2*^_*v*_ values were calculated using the *rsq* package in *R* [61]*.* We report *R*^*2*^_*v*_ values that have been adjusted for the number of predictors in each model (e.g., adj. *R*^*2*^_*v*_).

We also examined whether multicollinearity was present between predictor variables using Variance Inflation Factors (VIF). The VIFs evaluated on models that included individual activity frequencies and non-categorical demographic variables (e.g., age) ranged between 1.35 and 3.96 (*M* = 2.66), below the typical cut-offs of 5 or 10. Variables with the highest VIFs included participation frequency for poker in venues (3.96), eSports in venues (3.87), eSports online (3.48), poker online (3.46), casino card/table games in venues (3.46), and casino card/table games online (3.28). All other VIFs were < 3.00. We also examined the VIF for models that included the breadth of involvement online or in venues. The VIF for the breadth of online (13.58) and venue (17.34) involvement exceeded recommended cut-offs and were therefore excluded from the regression models.

In addition to the main regression analyses, we also performed a series of additional exploratory Quasi-Poisson regressions for each activity pair (e.g., online EGM and venue-based EGM). These analyses included the frequency of gambling on each activity pair, demographic variables, the breadth of involvement in online gambling, and the breadth of involvement in venue-based gambling. We summarize these results for these analyses in the main text, with the complete tables presented in the supplementary information. Relevant VIF scores for these analyses are presented in Additional file 1: Table S1 and the results of each regression are presented in Additional file 1: Tables S2-S15.

## Results

The demographic characteristics of the sample are presented in Table 1. A majority of participants identified as male, European, were married or in a defacto relationship, had listed their highest level of education as post-secondary school, were working full- or part-time, were born in Australia, and did not speak a language other than English at home. Participants were aged between 18 and 85 years. PGSI scores were strongly skewed (*M* = 3.91, *SD* = 5.56, *Mdn* = 1.00, Skew = 1.73, Kurtosis = 2.62, Min = 0, Max = 27), as were Kessler 6 scores (*M* = 5.64, *SD* = 6.14, *Mdn* = 3.00, Skew = 1.08, Kurtosis = 0.33, Min = 0, Max = 27).

### Gambling activity frequency

The frequency of participation in each online and venue gambling activity is presented in Table 2. When aggregating across each level of frequency, 76.75% of participants had gambled online using lottery activities, 50.10% on sports betting, 49.80% race wagering, and 43.95% on EGMs. In terms of venue-based gambling, 53.91% of participants had gambled on lottery activities, 35.57% on sports betting, 36.57% on race wagering, and 49.50% on EGMs. For other activities available online and in venues, responses were skewed towards not having participated in the last 4 weeks (e.g., > = 76.15% had not participated).

**Table 2:** Counts and percentages for frequency of participation in online and venue-based gambling sctivities

	Frequency of Activity			
	Not in the last 4 weeks	At least once in the last 4 weeks	At least once per week	At least once per day
Online Gambling Activity				
Lottery	232 (23.25%)	250 (25.05%)	453 (45.39%)	63 (6.31%)
EGM	563 (56.41%)	240 (24.05%)	167 (16.73%)	28 (2.81%)
Sports Betting	498 (49.90%)	183 (18.34%)	271 (27.15%)	46 (4.61%)
eSports betting	823 (82.46%)	83 (8.32%)	68 (6.81%)	24 (2.40%)
Race Wagering	501 (50.20%)	180 (18.04%)	260 (26.05%)	57 (5.71%)
Poker	760 (76.15%)	130 (13.03%)	84 (8.42%)	24 (2.40%)
Casino Games	761 (76.25%)	134 (13.43%)	80 (8.02%)	23 (2.30%)
Venue Gambling Activity				
Lottery	460 (46.09%)	202 (20.24%)	297 (29.76%)	39 (3.91%)
EGM	504 (50.50%)	253 (25.35%)	211 (21.14%)	30 (3.01%)
Sports Betting	643 (64.43%)	155 (15.53%)	175 (17.54%)	25 (2.51%)
eSports betting	836 (83.77%)	75 (7.52%)	68 (6.81%)	15 (1.50%)
Race Wagering	633 (63.43%)	173 (17.33%)	160 (16.03%)	32 (3.21%)
Poker	796 (79.76%)	112 (11.22%)	74 (7.41%)	16 (1.60%)
Casino Games	779 (78.06%)	130 (13.03%)	75 (7.52%)	14 (1.40%)

Associations between the frequencies of participation in each gambling activity were examined using a series of Spearman’s Rho correlations. As shown in Table 3, significant correlations were observed between most activities participated in online or in venues. Significant positive correlations were observed between online and venue participation frequency for the same activity pairs. That is, participants who frequently – or infrequently - gambled online using any particular activity (e.g., EGMs) were likely to also be frequently using – or infrequently using - that same activity type in venues.

**Table 3:** Bivariate Spearman’s Rho Correlations for Gambling Activity Frequencies

	1.	2.	3.	4.	5.	6.	7.	8.	9.	10.	11.	12.	13.
Online													
1. Lottery	–												
2. EGM	0.25^***^	–											
3. Sports betting	0.02	0.33^***^	–										
4. eSports betting	0.25^***^	0.47^***^	0.41^***^	–									
5. Race wagering	−0.02	0.26^***^	0.44^***^	0.29^***^	–								
6. Poker	0.22^***^	0.50^***^	0.38^***^	0.64^***^	0.28^***^	–							
7. Casino games	0.26^***^	0.53^***^	0.41^***^	0.63^***^	0.31^***^	0.74^***^	–						
Venue													
8. Lottery	**0.41** ^***^	0.42^***^	0.25^***^	0.35^***^	0.22^***^	0.35^***^	0.38^***^	–					
9. EGM	0.21^***^	**0.72** ^***^	0.32^***^	0.40^***^	0.30^***^	0.46^***^	0.45^***^	0.47^***^	–				
10. Sports betting	0.22^***^	0.47^***^	**0.66** ^***^	0.52^***^	0.38^***^	0.51^***^	0.55^***^	0.46^***^	0.44^***^	–			
11. eSports betting	0.25^***^	0.44^***^	0.37^***^	**0.82** ^***^	0.30^***^	0.61^***^	0.65^***^	0.39^***^	0.41^***^	0.54^***^	–		
12. Race wagering	0.14^**^	0.41^***^	0.44^***^	0.45^***^	**0.65** ^***^	0.42^***^	0.44^***^	0.42^***^	0.44^***^	0.62^***^	0.49^***^	–	
13. Poker	0.24^***^	0.50^***^	0.38^***^	0.67^***^	0.28^***^	**0.79** ^***^	0.69^***^	0.39^***^	0.46^***^	0.54^***^	0.71^***^	0.46^***^	–
14. Casino games	0.25^***^	0.51^***^	0.42^***^	0.61^***^	0.30^***^	0.67^***^	**0.78** ^***^	0.42^***^	0.49^***^	0.55^***^	0.68^***^	0.48^***^	0.73^***^

** = *p* < .01, *** = *p* < .001. Correlations between pairs of online and venue activities (e.g., online lottery and venue lottery) are bolded

Significant positive correlations were also observed between different activities in the same modality of access (e.g., online poker and online casino card/table games), and between different activities in different access modalities (e.g., online eSports betting and venue casino card/table games). This consistent pattern of positive correlations suggests that the frequency of gambling – or not gambling – on any particular activity is reflected in other activities regardless of modality. However, two pairs of correlations differed from the pattern described above. Participation frequency in gambling using online lottery-type activities was not significantly correlated with the participation frequency for online sports betting, or with the participation frequency for online race wagering.

### Gambling Frequency & Problem Gambling Severity

As shown in Table 4, PGSI scores were significantly and positively correlated with the frequency with which participants gambled online using lottery-type (*r*_*s*_(996) = 0.17, *p* < .001), EGM (*r*_*s*_(996) = 0.51, *p* < .001), sports betting (*r*_*s*_(996) = 0.31, *p* < .001), eSports betting (*r*_*s*_(996) = 0.41, *p* < .001), race wagering (*r*_*s*_(996) = 0.28, *p* < .001), poker (*r*_*s*_(996) = 0.41, *p* < .001), and casino card/table games (*r*_*s*_(996) = 0.44, *p* < .001). PGSI scores were also significantly and positively correlated with the frequency with which these same participants gambled in venues using lottery-type (*r*_*s*_(996) = 0.39, *p* < .001), EGM (*r*_*s*_(996) = 0.50, *p* < .001), sports betting (*r*_*s*_(996) = 0.43, *p* < .001), eSports betting (*r*_*s*_(996) = 0.41, *p* < .001), race wagering (*r*_*s*_(996) = 0.38, *p* < .001), poker (*r*_*s*_(996) = 0.43, *p* < .001), and casino card/table games (*r*_*s*_(996) = 0.43, *p* < .001).

**Table 4:** Median and 25th–75th percentile PGSI scores by gambling activity frequency

	Frequency of Activity			
	Not in the last 4 weeks	At least once in the last 4 weeks	At least once per week	At least once per day
Online Gambling Activity				
Lottery	1.00 [0.00, 3.00]	1.00 [0.00, 4.00]	2.00 [0.00, 7.00]	9.00 [2.00, 15.00]
EGM	0.00 [0.00, 2.00]	3.00 [0.00, 9.00]	8.00 [2.00, 12.00]	14.50 [9.00, 17.25]
Sports Betting	0.00 [0.00, 3.00]	2.00 [0.00, 8.50]	3.00 [0.00, 9.00]	9.00 [5.00, 14.00]
eSports betting	1.00 [0.00, 3.00]	7.00 [2.00, 11.00]	10.00 [5.75, 15.00]	14.50 [9.00, 17.25]
Race Wagering	0.00 [0.00, 3.00]	2.00 [0.00, 8.25]	3.00 [0.00, 9.00]	5.00 [1.00, 12.00]
Poker	1.00 [0.00, 3.00]	5.00 [1.00, 10.75]	9.00 [3.00, 14.00]	10.50 [8.75, 16.25]
Casino Games	1.00 [0.00, 3.00]	6.00 [1.25, 11.00]	9.00 [5.00, 13.00]	10.00 [8.00, 16.50]
Venue Gambling Activity				
Lottery	0.00 [0.00, 2.00]	2.00 [0.00, 8.00]	3.00 [1.00, 9.00]	11.00 [7.00, 17.00]
EGM	0.00 [0.00, 2.00]	2.00 [0.00, 6.00]	7.00 [2.00, 12.00]	11.50 [9.00, 17.00]
Sports Betting	0.00 [0.00, 2.00]	4.00 [1.00, 10.00]	7.00 [1.00, 11.00]	12.00 [9.00, 17.00]
eSports betting	1.00 [0.00, 3.00]	9.00 [3.50, 12.00]	10.00 [4.75, 16.00]	13.00 [8.50, 17.00]
Race Wagering	0.00 [0.00, 3.00]	3.00 [1.00, 10.00]	5.00 [1.00, 11.00]	9.00 [2.00, 17.00]
Poker	1.00 [0.00, 3.00]	7.50 [2.00, 11.00]	9.00 [5.00, 15.00]	11.50 [9.00, 17.50]
Casino Games	1.00 [0.00, 3.00]	5.00 [1.00, 11.00]	10.00 [8.00, 15.50]	14.50 [10.00, 17.00]

The 25th and 75th percentiles for each activity at each frequency level are presented in square brackets

However, these analyses do not account for the overlap between the frequency of gambling using different activity types or modalities (e.g., those who frequently gamble on sports online also frequently gambled on sports in venues), making it difficult to determine if problem gambling severity is uniquely associated with the frequency of gambling using any single activity online or in venues. To identify which - if any - activities and modalities were uniquely associated with problem gambling severity, we performed a Quasi-Poisson regression using PGSI scores as the dependent variable (e.g., [16, 51]). Separate independent variables were included for the frequency of gambling on each activity type online or in venues. To control for potential demographic effects on PGSI scores, age, gender, relationship status, education, household income, work status, and ethnicity were also included in the model (Table 5). The model accounted for a modest amount of variance in PGSI scores (Adj. *R*^*2*^_*v*_ = .34).

**Table 5:** Summary of quasi-poisson regression predicting PGSI scores

	*B*	SE	*t*	Lower CI	Upper CI	*p*
(Intercept)	0.79	0.32	2.46	0.16	1.41	.014
Online Gambling Frequency						
Lottery	−0.03	0.06	−0.57	−0.14	0.08	.567
EGM	**0.25**	**0.06**	**4.12**	**0.13**	**0.37**	**< .001**
Sports betting	−0.04	0.06	−0.70	− 0.16	0.07	.483
eSports betting	0.08	0.07	1.04	−0.07	0.22	.300
Race wagering	0.07	0.06	1.24	− 0.04	0.18	.217
Poker	−0.07	0.08	−0.94	− 0.23	0.08	.346
Casino games	0.00	0.07	0.05	−0.14	0.15	.963
Venue Gambling Frequency						
Lottery	0.03	0.06	0.61	−0.07	0.14	.539
EGM	**0.30**	**0.06**	**5.00**	**0.18**	**0.42**	**< .001**
Sports betting	**0.13**	**0.06**	**2.07**	**0.01**	**0.26**	**.039**
eSports betting	−0.03	0.08	−0.35	−0.19	0.13	.726
Race wagering	0.02	0.06	0.38	−0.10	0.14	.703
Poker	−0.04	0.08	−0.47	−0.21	0.13	.638
Casino games	0.16	0.08	1.96	−0.00	0.31	.050
Age	**−0.02**	**0.00**	**−4.83**	**−0.02**	**−0.01**	**< .001**
Gender (ref. Male)	0.08	0.08	0.97	−0.08	0.24	.331
Ethnicity (ref. European)						
Asian	**0.23**	**0.11**	**2.05**	**0.01**	**0.46**	**.040**
Other	0.09	0.16	0.52	−0.25	0.40	.603
Indigenous Australian	0.11	0.22	0.50	−0.34	0.51	.619
Relationship Status (ref. Not in a romantic relationship)						
Casually dating (i.e., not exclusive)	−0.00	0.17	−0.01	−0.34	0.32	.995
Exclusively dating	−0.03	0.17	−0.19	−0.37	0.29	.850
Engaged	0.23	0.22	1.01	−0.23	0.64	.311
Living together	0.13	0.15	0.90	−0.16	0.41	.368
Married or defacto	0.09	0.10	0.96	−0.09	0.28	.335
Education (ref. Postgraduate qualification)						
University or college degree	−0.05	0.12	−0.42	−0.28	0.19	.672
Trade/technical certificate/diploma	0.01	0.14	0.06	−0.26	0.28	.956
Year 12 or equivalent	−0.05	0.14	−0.32	−0.32	0.23	.746
Year 10 or less	0.03	0.17	0.19	−0.31	0.37	.849
Work Status (ref. Work full-time)						
Work part-time or casual	0.14	0.11	1.33	−0.07	0.34	.185
Full-time student	0.08	0.18	0.46	−0.27	0.42	.647
Full-time home duties	0.07	0.16	0.41	−0.26	0.38	.681
Unemployed	−0.19	0.13	−1.46	−0.45	0.06	.146
Household Income (ref. Less than $25,000 per year)						
$25,000–$49,999	**− 0.31**	**0.15**	**−2.02**	**−0.61**	**−0.01**	**.043**
$50,000–$74,999	−0.31	0.16	−1.90	−0.62	0.01	.058
$75,000–$99,999	**−0.51**	**0.17**	**−2.96**	**−0.84**	**−0.17**	**.003**
$100,000–$124,999	**−0.54**	**0.19**	**−2.85**	**−0.90**	**− 0.17**	**.004**
$125,000–$149,999	**− 0.42**	**0.20**	**−2.07**	**− 0.81**	**− 0.02**	**.039**
> $150,000	− 0.34	0.19	−1.83	− 0.70	0.02	.067
I prefer not to say	**−0.62**	**0.19**	**−3.19**	**−1.00**	**−0.24**	**.001**
Other Language at Home (ref. Yes)	−0.00	0.12	−0.04	− 0.25	0.24	.968

Adj. *R*^*2*^_v_ = .34. Bolding of model parameters indicates that the coefficient was a significant unique predictor. Levels within categorical predictors were collapsed when there were only a small proportion of participants that endorsed an option (e.g., Ethnicity – Other includes Middle Eastern, African, Latin, Central and South American, and Pacific Islander participants). A single participant selected “Other” as their gender and was omitted from the regression

When controlling for all other variables, the frequency of online gambling using EGMs, venue gambling using EGMs, and venue gambling using sports betting each uniquely predicted greater PGSI scores. Demographic variables were also observed to be unique predictors of PGSI scores. Identification as South-East, East, or South Asian uniquely predicted greater PGSI scores, relative to identification as European. Conversely, an older age uniquely predicted smaller PGSI scores, as did being unwilling to report a household income, or reporting a household income of $25,000–$49,999, $75,000–$99,999, $100,000–$124,999, and $125,000–$149,999, relative to an income of less than $25,000 per year.

### Gambling frequency and psychological harms

As with problem gambling severity, psychological distress was positively correlated with gambling frequency online and in venues (Table 6). Significant positive correlations were observed for online gambling on lottery type activities (*r*_*s*_(996) = 0.14, *p* < .001), EGMs (*r*_*s*_(996) = 0.32, *p* < .001), sports betting (*r*_*s*_(996) = 0.18, *p* < .001), eSports betting (*r*_*s*_(996) = 0.26, *p* < .001), poker (*r*_*s*_(996) = 0.29, *p* < .001), and casino card/table games (*r*_*s*_(996) = 0.29, *p* < .001), but not race wagering (*r*_*s*_(996) = 0.05, *p* = 1.000). Psychological distress was also significantly positively correlated with the frequency of gambling in venues for lottery-type (*r*_*s*_(996) = 0.24, *p* < .001), EGMs (*r*_*s*_(996) = 0.29, *p* < .001), sports betting (*r*_*s*_(996) = 0.30, *p* < .001), eSports betting (*r*_*s*_(996) = 0.26, *p* < .001), race wagering (*r*_*s*_(996) = 0.17, *p* < .001), poker (*r*_*s*_(996) = 0.28, *p* < .001), and casino card/table games (*r*_*s*_(996) = 0.30, *p* < .001).

**Table 6:** Median and 25th–75th percentile kessler 6 scores by gambling activity frequency

	Frequency of Activity			
	Not in the last 4 weeks	At least once in the last 4 weeks	At least once per week	At least once per day
Online Gambling Activity				
Lottery	2.00 [0.00, 7.00]	4.00 [1.00, 9.00]	4.00 [0.00, 11.00]	8.00 [1.00, 15.50]
EGM	2.00 [0.00, 6.00]	6.00 [2.00, 12.00]	7.00 [2.50, 12.50]	12.00 [3.75, 16.25]
Sports Betting	2.00 [0.00, 7.00]	5.00 [2.00, 11.00]	4.00 [0.00, 12.00]	8.50 [3.00, 15.00]
eSports betting	3.00 [0.00, 7.00]	7.00 [2.00, 12.00]	12.00 [5.75, 14.00]	12.00 [3.50, 17.00]
Race Wagering	3.00 [0.00, 7.00]	5.00 [1.00, 12.00]	3.00 [0.00, 11.00]	5.00 [0.00, 12.00]
Poker	2.00 [0.00, 7.00]	7.00 [3.00, 13.00]	10.00 [3.00, 13.00]	12.50 [6.00, 17.25]
Casino Games	3.00 [0.00, 7.00]	6.00 [2.00, 12.75]	12.00 [6.00, 15.00]	12.00 [3.50, 17.00]
Venue Gambling Activity				
Lottery	2.00 [0.00, 6.00]	5.00 [1.00, 11.00]	6.00 [1.00, 12.00]	11.00 [3.00, 16.00]
EGM	2.00 [0.00, 6.00]	5.00 [1.00, 11.00]	6.00 [2.00, 12.00]	12.00 [8.00, 16.00]
Sports Betting	2.00 [0.00, 6.00]	8.00 [2.00, 12.00]	7.00 [1.00, 12.00]	12.00 [3.00, 17.00]
eSports betting	3.00 [0.00, 7.00]	11.00 [3.00, 13.00]	11.00 [3.75, 13.25]	13.00 [7.00, 18.00]
Race Wagering	3.00 [0.00, 7.00]	6.00 [2.00, 12.00]	6.00 [0.00, 12.00]	5.50 [0.75, 16.50]
Poker	3.00 [0.00, 7.00]	8.50 [2.75, 13.00]	11.00 [4.25, 13.00]	14.00 [7.75, 18.25]
Casino Games	3.00 [0.00, 7.00]	6.00 [2.00, 13.00]	12.00 [6.00, 13.50]	16.50 [9.00, 18.75]

The 25th and 75th percentiles for each activity at each frequency level are presented in square brackets

A Quasi-Poisson regression model was used to examine which activities uniquely predicted psychological distress. The same independent variables - including demographics - used in the PGSI model were used in the Kessler 6 model (Table 7). The model accounted for a small amount of variance in Kessler 6 scores (adj. *R*^*2*^_*v*_ = .16). When controlling for all other variables, higher frequencies of venue gambling using EGMs, sports betting, and casino card/table games were uniquely associated with increased psychological distress. Greater age was uniquely associated with decreased psychological distress. That is, older adults had less psychological distress than younger adults.

**Table 7:** Summary of quasi-poisson regression predicting kessler 6 scores

	*B*	SE	*t*	Lower CI	Upper CI	*p*
(Intercept)	1.79	0.29	6.24	1.23	2.36	< .001
Online Gambling Frequency						
Lottery	0.05	0.05	1.12	−0.04	0.14	.261
EGM	0.08	0.06	1.50	− 0.03	0.19	.135
Sports betting	− 0.06	0.05	−1.10	− 0.16	0.05	.273
eSports betting	0.00	0.07	0.03	−0.14	0.14	.975
Race wagering	−0.04	0.05	−0.72	−0.13	0.06	.471
Poker	0.06	0.07	0.89	−0.08	0.20	.373
Casino games	0.02	0.07	0.26	−0.12	0.16	.797
Venue Gambling Frequency						
Lottery	0.02	0.05	0.43	−0.07	0.11	.665
EGM	**0.12**	**0.05**	**2.20**	**0.01**	**0.23**	**.028**
Sports betting	**0.15**	**0.06**	**2.54**	**0.03**	**0.27**	**.011**
eSports betting	−0.07	0.08	−0.89	−0.23	0.09	.375
Race wagering	−0.04	0.06	−0.73	−0.16	0.07	.467
Poker	−0.08	0.08	−1.01	−0.24	0.08	.314
Casino games	**0.17**	**0.08**	**2.15**	**0.01**	**0.31**	**.032**
Age	**−0.02**	**0.00**	**−5.10**	**−0.02**	**− 0.01**	**< .001**
Gender (ref. Male)	0.03	0.07	0.35	−0.12	0.17	.730
Ethnicity (ref. European)						
Asian	0.02	0.11	0.19	−0.20	0.24	.851
Other	0.06	0.15	0.42	−0.24	0.34	.675
Indigenous Australian	0.18	0.19	0.94	−0.22	0.54	.350
Relationship Status (ref. Not in a romantic relationship)						
Casually dating (i.e., not exclusive)	−0.00	0.15	−0.01	−0.31	0.29	.991
Exclusively dating	−0.03	0.15	−0.19	−0.33	0.26	.848
Engaged	−0.01	0.23	−0.05	−0.49	0.42	.962
Living together	−0.18	0.14	−1.33	−0.45	0.08	.185
Married or defacto	−0.15	0.08	−1.88	−0.31	0.01	.061
Education (ref. Postgraduate qualification)						
University or college degree	0.15	0.12	1.30	−0.07	0.38	.193
Trade/technical certificate/diploma	0.08	0.13	0.65	−0.16	0.33	.514
Year 12 or equivalent	0.17	0.13	1.28	−0.09	0.43	.201
Year 10 or less	0.14	0.15	0.92	−0.16	0.44	.357
Work Status (ref. Work full-time)						
Work part-time or casual	−0.00	0.09	−0.01	−0.19	0.18	.995
Full-time student	−0.29	0.17	−1.65	−0.64	0.04	.098
Full-time home duties	−0.17	0.16	−1.07	−0.48	0.13	.283
Unemployed	−0.03	0.11	−0.27	−0.24	0.18	.790
Household Income (ref. Less than $25,000 per year)						
$25,000–$49,999	−0.19	0.14	−1.37	−0.46	0.09	.171
$50,000–$74,999	0.06	0.14	0.44	−0.22	0.35	.659
$75,000–$99,999	−0.14	0.15	−0.91	−0.44	0.16	.363
$100,000–$124,999	−0.28	0.17	−1.67	−0.61	0.05	.095
$125,000–$149,999	−0.09	0.18	−0.48	−0.44	0.27	.634
> $150,000	−0.14	0.17	−0.79	−0.48	0.20	.432
I prefer not to say	−0.27	0.17	−1.63	−0.60	0.06	.104
Other Language at Home (ref. Yes)	0.03	0.12	0.27	−0.20	0.27	.791

Adj. *R*^*2*^_*v*_ = .13. Bolding of model parameters indicates that the coefficient was a significant unique predictor. Levels within categorical predictors were collapsed when there were only a small proportion of participants that endorsed an option (e.g., Ethnicity – Other includes Middle Eastern, African, Latin, Central and South American, and Pacific Islander participants). A single participant selected “Other” as their gender and was omitted from the regression

### Breadth of involvement, problem gambling severity, & psychological harms

We examined whether the breadth of online or venue gambling was associated with PGSI and K6 scores. Participants engaged in between 1 to 7 activities online (*Mdn* = 2, 25th percentile = 1, 75th percentile = 4), and between 0 to 7 in venues (*Mdn* = 2, 25th percentile = 0, 75th percentile = 4). The breadth of online gambling was significantly positively correlated with the breadth of venue gambling (*r*_*s*_(996) = 0.79, *p* < .001). Online (*r*_*s*_(996) = 0.52, *p* < .001) and venue (*r*_*s*_(996) = 0.55, *p* < .001) breadth were both significantly positively correlated with PGSI scores. Similarly, both online (*r*_*s*_(996) = 0.32, *p* < .001) and venue (*r*_*s*_(996) = 0.34, *p* < .001) breadth were both significantly positively correlated with K6 scores.

As noted in the method, we performed a series of exploratory Quasi-Poisson regressions for each activity pair while statistically controlling for breadth of involvement in online- and venue-based gambling. The complete results of the Quasi-Poisson regressions for PGSI scores are presented in Additional file 1: Tables S2-S8 and the complete results of the Quasi-Poisson regressions for K6 scores are presented in Additional file 1: Tables S9-S15. In general, these regressions yielded a similar pattern of results as the main analyses and accounted for a similar proportion of variance in PGSI (Adj. *R*^*2*^_*v*_ = 0.32–0.36) and Kessler 6 (Adj. *R*^*2*^_*v*_ = 0.15) scores. The frequency of online and venue EGM gambling remained significant predictors of PGSI scores, even when controlling for breadth of gambling involvement, and each other. However, neither venue-based EGM play or venue-based casino card/table games were unique predictors of K6 scores. Venue-based sports betting was no longer a unique predictor of PGSI scores, or of K6 scores.

The breadth of online gambling involvement or venues gambling involvement was generally associated with PGSI scores, but inconsistently associated with K6 scores. Both online and venue-based breadth of gambling involvement were positively associated with PGSI scores, except when controlling for online and venue-based EGM play, as shown in Additional file 1: Table S2. Associations between breadth of gambling and the K6 were inconsistent. Neither online or venue breadth of involvement were associated with K6 scores when controlling for EGM or casino game play. Venue – but not online – breadth was associated with K6 scores when controlling for lottery, eSports, and poker involvement. Conversely, online – but not venue – breadth predicted K6 scores when controlling for sports betting. Both online and venue breadth predicted K6 scores when only controlling for race wagering.

## Discussion

In this study, we aimed to isolate the impact of specific gambling activities and modalities to advance our understanding of the relationship between gambling participation and problem gambling severity, and psychological distress. As anticipated, we found that frequency of participation in each gambling activity and modality was associated with greater problem gambling severity and psychological distress. When controlling for demographic variables and overlap between participation across activities, we found that the frequency of specific gambling activities and modalities were related to greater reported gambling problem severity and psychological distress in a sample of past-month internet gamblers. Critically, because the measures of gambling frequency included an option for non-participation, by controlling for each activity type we inherently controlled for breadth of participation.

We found that those who engaged in an online version of a gambling activity were likely to have also engaged in the offline activity. This is consistent with previous research suggesting a positive and complementary relationship between online and offline gambling activity [62, 63]. Previous research on motivations for Internet gambling suggest that although convenience and accessibility are the predominant factors in choosing this channel, many Internet gamblers will still participate in offline gambling [30, 31]. Note, however, that we cannot speak to the causal direction of the relationship between online and offline gambling and future research should examine the temporal sequence of engagement with gambling activities and modes and development of problems. It is unclear whether engagement in an online activity may motivate the uptake of the offline variance or vice-verse – or if the two are not causally related at all, and our data are unable to speak to this question.

Our results indicate that despite strong correlations between the frequency of play in each modality, specific activities online and in venues were uniquely associated with the severity of problem gambling or psychological distress. As hypothesised, the frequency of participation in EGM online and in venues uniquely predicted greater problem gambling severity scores, even when controlling for the frequency of gambling on other activities. This is consistent with previous research and theory suggesting a strong relationship between the use of EGMs and the experience of gambling-related problems [33–35]. Notably, our finding replicates and extends on previous observations that EGM use – particularly venue-based – is strongly associated with gambling problems even when controlling for the overall breadth of gambling involvement [32]. The finding that both online and venue-based EGMs were independently related to gambling problems suggests that there may be something about the game itself that is problematic, for example, the short interval between bets and outcomes enabling rapid, continuous periods of betting. However, the finding that only venue-based EGM participation was uniquely related to greater psychological distress suggests that there are differences between the modalities of access. Further research is needed to determine if there are differences between online and venue-based EGMs or various types of EGMs that may moderate the relation between frequent participation and the experience of gambling problems and psychological harms.

Contrary to public debate surrounding online sports betting in Australia, participation in venue-based sports betting was uniquely associated with greater problem gambling severity scores and psychological distress, even when controlling for online sports betting. Given the additional effort needed to visit venues rather than placing sports bets online, Internet gamblers who also gamble in person may be more intensely involved in this activity. Our findings are contrary to previous Australian research that indicate that sports betting was associated with problems among Internet, but not land-based gamblers [2, 28]. However, these previous results were not specific in that the Internet gamblers with problems may have been using land-based venues for their sports betting. Further, our sample did not include exclusively land-based gamblers and this relationship should be investigated in a broader population of gamblers.

Unique to this study is the finding that certain gambling activities were related to distress, but not gambling problems. Specifically, involvement in venue-based table and card games were uniquely associated with greater levels of psychological distress, but not problem gambling severity. Within the Australian context, casino and card games are only available from offshore gambling sites and land-based casinos; which are mostly limited to major Australian cities. The Pathways Model of problem gambling [20] presents emotional vulnerability as a risk factor for developing a gambling disorder and there is substantial evidence that psychological distress and mental health disorders, including anxiety and depression, are a risk factor for the experience of gambling problems [4, 45]. Individuals experiencing psychological distress may engage in gambling in an attempt to escape or negate these emotions [64]. Not all individuals who use gambling to cope with distress will develop gambling problems, and this may be moderated by the type of gambling they use. These findings may indicate that casino and card games have a lower potential to lead to problems, even among those psychologically vulnerable. Given the relatively limited availability, participation in casino-based gambling (excluding EGMs) is not often the focus of harm-minimisation efforts or campaigns. Although our cross-sectional results cannot indicate that those with higher rates of psychological distress are at risk of later developing gambling problems, they are still an important subgroup to consider in terms of policies to minimise harms. These findings should be replicated in a sample including non-Internet gamblers, as it may be that Internet gamblers have lower levels of interest in casino and card games in land-based venue or are less able to access these.

### Implications

Our findings support the emphasis placed on EGMs as a predominant component in the experience of gambling problems in Australia, but also broaden the current focus to include online variants, only accessible through offshore gambling sites. The findings that more popular activities (e.g., lotteries) had a lower association with problems supports the association between greater overall gambling participation and harms. That is, those engaged in more specialised gambling may be more immersed in gambling as an activity. Public health policies may therefore target specialised gambling venues rather than the broad community to reach the most relevant cohorts. A broader range of gambling activities were uniquely related to psychological distress. In general, our results suggest that an increased focus on accurately identifying the cause, direction, and boundary conditions of these relationships is needed in empirical research.

Additionally, our results confirm that there are at-risk subgroups within the population of Australian Internet gamblers. Specifically, younger adults were more likely to experience greater gambling problems and psychological distress. This is consistent with previous research finding younger age groups are at-risk of experiencing gambling problems [2, 42, 65, 66], but confirms that this is independent of participation in any specific activity, or overall gambling involvement. This suggests that there is something about younger age that is related to problematic gambling, potentially the increased propensity for risk taking and reduced awareness or consideration of potential negative consequences [67, 68]. However, it should be noted that our sample was more likely to include those aged 30 to 65; it is possible that there is a response bias and younger panel members may not be representative of young gamblers in general.

### Limitations

As with any study there are important limitations to consider when interpreting the results. First, the data are cross-sectional, and we cannot make any causal inferences. Our results cannot distinguish between participants who are experiencing gambling problems and/or psychological distress and are motivated to preferentially gamble using particular activities/use certain modalities, and participants who experience problems and/or distress because of their gambling using one activity or modality over another.

Second, the sample is not representative of the broad population of gamblers. Participants self-selected from an existing panel held by a market research company and included only those who had gambled online in the past 30 days to focus on those who gamble online regularly. As participants received a small payment for participation, it is possible that false responses were made that were undetected. The panel provider did not disclose the response rate; future research using panels should ensure that market research companies are more transparent about data collection. It is possible that land-based (non-Internet) gamblers may have different patterns of gambling involvement which lead to harms that would not be detected in this research. Moreover, the results we report may be specific to the Australian regulatory context. As noted above, many of the online gambling activities evaluated in the present study were only accessible using offshore gambling operators. Previous research has found that Australian participants who used offshore gambling operators reported greater problem gambling severity and gambling involvement than those who used domestic operators [69]. Many participants in the present study may be drawn from a similar sub-group of Internet gamblers and are therefore unlikely to be representative of Australian internet gamblers, or gamblers in general. Due to the limitations of the Qualtrics platform, we are unable to calculate a response rate, and as such the sample may not be fully representative of even Australian Internet gamblers.

Third, the current study used the PGSI as a measure of problem gambling severity. This measure has been criticised and may not accurately measure all gambling-related harms [70–72]. Unlike past studies we did not use the original [50] or alternative [52] cut-offs for the PGSI. We are therefore limited in the inferences we can make regarding the extent that specific activities and/or modalities uniquely predict membership to a specific PGSI category.

Fourth, in the present study we used frequency of gambling as a proxy for involvement. This approach is coarse-grained, and overlooks the combined impact of frequency of gambling, expenditure on gambling, and disposable income on gambling problems and psychological distress. By conceptualising gambling involvement as the frequency of gambling, there is the possibility that different patterns of gambling behaviour – and their relationship with problem gambling and psychological distress – are obscured. For example, an individual with a large expenditure on sports once a month may be at more – or less – risk of developing gambling problems and psychological distress than an individual with a small expenditure on EGMs once per day. The dataset used in the present study only assessed expenditure on online activities, and not land-based activities, and as such we are unable to distinguish between these two quite different hypothetical patterns.

Fifth, the fixed-response options used to measure frequency of gambling on each activity may introduce error to the reported estimates. While this ambiguity of response options and measurement is not limited to our study, it does limit the conclusions that can be made. The relationship between frequency of gambling on any given activity and PGSI/K6 scores could conceivably be stronger if intense but sporadic patterns of play are mistakenly measured as only being infrequent. The development of appropriate indices of self-reported gambling behaviour is difficult but will be necessary for accurate measurements when actual behavioural data is unavailable.

Sixth, the presence of correlations between activity frequencies may have contributed to suppression of some regression coefficients. This potential collinearity is owing to intrinsic overlap between online and venue participation (or non-participation). That is, some level of collinearity is unavoidable because of multimodal patterns of play. For researchers interested in controlling for multimodal behaviour at the activity level, there are limited methods for satisfactory addressing this limitation. While the application of data-reduction methods such as PCA will reduce the number of variables to non-related components, this also has the side effect of clustering together activities that differ structurally - and potentially at a modality level - due to similarities in participant responses. Other approaches such as dropping variables are similarly limited.

Future research may investigate the specific temporal relationships between gambling activities and modalities, such as whether there is a gateway effect between any activity and gambling problems. This may involve longitudinal research and should include samples that include non-Internet gamblers. Of interest from a policy standpoint is whether the availability of specific forms of gambling changes, whether individuals migrate to alternate forms, or whether problems are reduced. It is important to note that associations between gambling activities and problem gambling severity are not necessarily fixed or stable over time. As changes are made to various forms of gambling, for example, changes to structural design and characteristics of play, this will likely impact the potential contribution of this form to the development of gambling problems. Similarly, the context in which gambling is available and presence of consumer protection resources may moderate this impact.

## Conclusion

The current research provides an important contribution to the understanding of the relationship between gambling participation in specific forms and modalities and problem gambling severity and psychological distress in Australian Internet gamblers. The results replicate previous studies indicating that frequency of gambling involvement is related to greater gambling problems and psychological distress. However, when controlling for demographic factors and involvement in other gambling activities and modalities, only participation in land-based and online EGMs and land-based sports betting were uniquely predictive of greater gambling problems. Our results suggest that among pas-month Internet gamblers, participation in Internet gambling in general is not uniquely related to greater gambling problems, and that a continued focus on EGMS in their various forms and modalities, is important to reduce gambling-related harms.

## Supplementary information

**Additional file 1.** Supplementary Methods and Results. These analyses separately test the relationship between frequency of gambling on a particular activity, while controlling for composite measures of gambling involvement.

## Data Availability

The dataset analysed in the current study is not publicly available, or available on reasonable request because participants explicitly consented to only have their data shared with the immediate research team.

## Abbreviations

EGMs: Electronic Gaming Machines
K6: Kessler 6
PGSI: Problem Gambling Severity Index
VIF: Variance Inflation Factors
