# Supplementary material for: Isolating the impact of specific gambling activities and modes on problem gambling and psychological distress in internet gamblers
Source: BMC Public Health. 2019 Oct 25;19:1372. doi: 10.1186/s12889-019-7738-5 (PMC6815058; doi:10.1186/s12889-019-7738-5)
Supplement: Supplementary file 1 — Additional file 1. Supplementary Methods and Results. These analyses separately test the relationship between frequency of gambling on a particular activity, while controlling for composite measures of gambling involvement. [file 12889_2019_7738_MOESM1_ESM.docx]

**Supplementary Method**

To allow for comparison with previous studies, we also performed a series of Quasi-Poisson regressions for each modality pair (e.g., online and venue variants of an activity). Unlike the regressions reported in the main manuscript, these included discrete variables for the breath of gambling online and the breath of gambling in venues. Adjusted *R^2^_v_* were also calculated for each model. We also examined collinearity for each regression, calculating VIFs for each set of predictor variables (e.g., online EGM frequency, venue EGM frequency, number of activities online, number of activities in venues, and age). These results are summarized in Table S1. Note that the VIFs for online and venue counts often exceeded 5, indicating the presence of multicollinearity.

*Table S1: Variance Inflation Factors for Individual Activity Regressions*

|  | EGM | Lottery | Sports Betting | eSports Betting | Race Wagering | Poker | Casino Games |
| --- | --- | --- | --- | --- | --- | --- | --- |
| Online Frequency | 2.55 | 1.39 | 2.18 | 3.53 | 1.96 | 3.29 | 3.13 |
| Venue Frequency | 2.52 | 2.23 | 2.99 | 3.62 | 2.66 | 3.47 | 3.23 |
| Online Activities Count | 4.94 | 4.86 | 5.04 | 4.82 | 4.76 | 5.15 | 5.36 |
| Venue Activities Count | 5.14 | 6.28 | 5.53 | 4.98 | 5.59 | 4.91 | 5.18 |
| Age | 1.21 | 1.22 | 1.21 | 1.21 | 1.29 | 1.20 | 1.20 |

Note: VIFs for each variable are reported in each row, while columns indicate the gambling activity used in that regression.

**Supplementary Results**

The Quasi-Poisson regressions for PGSI scores are presented in Tables S2-S8, and the regressions for K6 scores presented in Tables S9-S15. For all tables, significant unique predictors are bolded.

*Table S2: Summary of Quasi-Poisson Regression Predicting PGSI Scores for EGM Frequency and Involvement Breadth*

|  | *B* | SE | *t* | Lower CI | Upper CI | *p* |
| --- | --- | --- | --- | --- | --- | --- |
| **(Intercept)** | 0.92 | 0.31 | 3.01 | 0.32 | 1.52 | .003 |
| **Online EGM Frequency** | **0.24** | **0.06** | **3.87** | **0.12** | **0.36** | **< .001** |
| **Venue EGM Frequency** | **0.26** | **0.06** | **4.29** | **0.14** | **0.38** | **< .001** |
| **Online Activities Count** | 0.03 | 0.04 | 0.64 | -0.05 | 0.11 | .521 |
| **Venue Activities Count** | **0.10** | **0.04** | **2.69** | **0.03** | **0.17** | **.007** |
| **Age** | **-0.02** | **0.00** | **-4.40** | **-0.02** | **-0.01** | **< .001** |
| **Gender (ref. Male)** | 0.06 | 0.08 | 0.77 | -0.10 | 0.22 | .443 |
| **Ethnicity (ref. European)** |  |  |  |  |  |  |
| *Asian* | 0.20 | 0.11 | 1.75 | -0.03 | 0.42 | .081 |
| *Other* | 0.07 | 0.16 | 0.44 | -0.26 | 0.38 | .661 |
| *Indigenous Australian* | 0.08 | 0.22 | 0.38 | -0.37 | 0.49 | .701 |
| **Relationship Status (ref. Not in a romantic relationship)** |  |  |  |  |  |  |
| *Casually dating (i.e., not exclusive)* | -0.05 | 0.17 | -0.28 | -0.39 | 0.27 | .782 |
| *Exclusively dating* | -0.06 | 0.17 | -0.34 | -0.40 | 0.26 | .733 |
| *Engaged* | 0.24 | 0.22 | 1.07 | -0.22 | 0.65 | .284 |
| *Living together* | 0.12 | 0.15 | 0.79 | -0.18 | 0.40 | .427 |
| *Married or defacto* | 0.07 | 0.10 | 0.75 | -0.12 | 0.26 | .454 |
| **Education (ref. Postgraduate qualification)** |  |  |  |  |  |  |
| *University or college degree* | -0.03 | 0.12 | -0.26 | -0.26 | 0.21 | .796 |
| *Trade/technical certificate/diploma* | 0.03 | 0.14 | 0.22 | -0.24 | 0.30 | .826 |
| *Year 12 or equivalent* | -0.04 | 0.14 | -0.29 | -0.31 | 0.24 | .774 |
| *Year 10 or less* | 0.10 | 0.17 | 0.58 | -0.24 | 0.44 | .560 |
| **Work Status (ref. Work full-time)** |  |  |  |  |  |  |
| *Work part-time or casual* | 0.11 | 0.10 | 1.03 | -0.10 | 0.31 | .302 |
| *Full-time student* | 0.05 | 0.18 | 0.26 | -0.31 | 0.38 | .794 |
| *Full-time home duties* | 0.05 | 0.16 | 0.30 | -0.28 | 0.36 | .762 |
| *Unemployed* | -0.20 | 0.13 | -1.49 | -0.46 | 0.06 | .138 |
| **Household Income (ref. Less than $25,000 per year)** |  |  |  |  |  |  |
| *$25,000-$49,999* | -0.26 | 0.15 | -1.68 | -0.56 | 0.05 | .093 |
| *$50,000-$74,999* | -0.24 | 0.16 | -1.49 | -0.56 | 0.08 | .137 |
| *$75,000-$99,999* | **-0.49** | **0.17** | **-2.86** | **-0.82** | **-0.15** | **.004** |
| *$100,000-$124,999* | **-0.56** | **0.19** | **-3.02** | **-0.93** | **-0.20** | **.003** |
| *$125,000-$149,999* | **-0.43** | **0.20** | **-2.15** | **-0.82** | **-0.04** | **.032** |
| *>$150,000* | -0.31 | 0.18 | -1.67 | -0.67 | 0.05 | .095 |
| *I prefer not to say* | **-0.55** | **0.19** | **-2.82** | **-0.93** | **-0.17** | **.005** |
| **Other Language at Home (ref. Yes)** | -0.06 | 0.12 | -0.53 | -0.30 | 0.18 | .596 |

Note: Adj. *R^2^_v_* = .36. Bolding of model parameters indicates that the coefficient was a significant unique predictor. Levels within categorical predictors were collapsed when there were only a small proportion of participants that endorsed an option (e.g., Ethnicity – Other includes Middle Eastern, African, Latin, Central and South American, and Pacific Islander participants). A single participant selected “Other” as their gender and was omitted from the regression.

*Table S3: Summary of Quasi-Poisson Regression Predicting PGSI Scores for Lottery Frequency and Involvement Breadth*

|  | *B* | SE | *t* | Lower CI | Upper CI | *p* |
| --- | --- | --- | --- | --- | --- | --- |
| **(Intercept)** | 1.23 | 0.32 | 3.81 | 0.59 | 1.85 | < .001 |
| **Online Lottery Frequency** | 0.01 | 0.06 | 0.19 | -0.11 | 0.13 | .847 |
| **Venue Lottery Frequency** | 0.08 | 0.06 | 1.28 | -0.04 | 0.21 | .200 |
| **Online Activities Count** | **0.11** | **0.04** | **2.69** | **0.03** | **0.20** | **.007** |
| **Venue Activities Count** | **0.13** | **0.04** | **3.11** | **0.05** | **0.20** | **.002** |
| **Age** | **-0.01** | **0.00** | **-4.05** | **-0.02** | **-0.01** | **< .001** |
| **Gender (ref. Male)** | 0.07 | 0.08 | 0.88 | -0.09 | 0.24 | .382 |
| **Ethnicity (ref. European)** |  |  |  |  |  |  |
| *Asian* | 0.18 | 0.12 | 1.51 | -0.06 | 0.41 | .132 |
| *Other* | 0.04 | 0.17 | 0.26 | -0.31 | 0.37 | .798 |
| *Indigenous Australian* | 0.08 | 0.23 | 0.36 | -0.40 | 0.51 | .716 |
| **Relationship Status (ref. Not in a romantic relationship)** |  |  |  |  |  |  |
| *Casually dating (i.e., not exclusive)* | 0.08 | 0.18 | 0.46 | -0.28 | 0.41 | .643 |
| *Exclusively dating* | -0.05 | 0.18 | -0.30 | -0.41 | 0.28 | .762 |
| *Engaged* | 0.34 | 0.24 | 1.44 | -0.15 | 0.78 | .152 |
| *Living together* | 0.13 | 0.16 | 0.82 | -0.19 | 0.43 | .412 |
| *Married or defacto* | 0.05 | 0.10 | 0.53 | -0.15 | 0.26 | .600 |
| **Education (ref. Postgraduate qualification)** |  |  |  |  |  |  |
| *University or college degree* | -0.02 | 0.13 | -0.13 | -0.26 | 0.24 | .896 |
| *Trade/technical certificate/diploma* | 0.08 | 0.14 | 0.54 | -0.20 | 0.36 | .588 |
| *Year 12 or equivalent* | 0.03 | 0.15 | 0.19 | -0.26 | 0.32 | .848 |
| *Year 10 or less* | 0.21 | 0.18 | 1.15 | -0.15 | 0.56 | .250 |
| **Work Status (ref. Work full-time)** |  |  |  |  |  |  |
| *Work part-time or casual* | 0.12 | 0.11 | 1.06 | -0.10 | 0.33 | .288 |
| *Full-time student* | 0.06 | 0.19 | 0.31 | -0.32 | 0.42 | .758 |
| *Full-time home duties* | 0.11 | 0.17 | 0.62 | -0.24 | 0.43 | .534 |
| *Unemployed* | -0.18 | 0.14 | -1.27 | -0.45 | 0.09 | .204 |
| **Household Income (ref. Less than $25,000 per year)** |  |  |  |  |  |  |
| *$25,000-$49,999* | -0.26 | 0.16 | -1.58 | -0.57 | 0.07 | .115 |
| *$50,000-$74,999* | -0.21 | 0.17 | -1.20 | -0.54 | 0.14 | .232 |
| *$75,000-$99,999* | **-0.48** | **0.18** | **-2.65** | **-0.83** | **-0.12** | **.008** |
| *$100,000-$124,999* | **-0.60** | **0.20** | **-3.06** | **-0.99** | **-0.22** | **.002** |
| *$125,000-$149,999* | **-0.42** | **0.21** | **-2.00** | **-0.84** | **-0.01** | **.046** |
| *>$150,000* | -0.20 | 0.19 | -1.04 | -0.58 | 0.18 | .301 |
| *I prefer not to say* | **-0.57** | **0.21** | **-2.74** | **-0.98** | **-0.17** | **.006** |
| **Other Language at Home (ref. Yes)** | -0.05 | 0.13 | -0.39 | -0.30 | 0.21 | .699 |

Note: Adj. *R^2^_v_* = .32. Bolding of model parameters indicates that the coefficient was a significant unique predictor. Levels within categorical predictors were collapsed when there were only a small proportion of participants that endorsed an option (e.g., Ethnicity – Other includes Middle Eastern, African, Latin, Central and South American, and Pacific Islander participants). A single participant selected “Other” as their gender and was omitted from the regression.

*Table S4: Summary of Quasi-Poisson Regression Predicting PGSI Scores for Sports Betting Frequency and Involvement Breadth*

|  | *B* | SE | *t* | Lower CI | Upper CI | *p* |
| --- | --- | --- | --- | --- | --- | --- |
| **(Intercept)** | 1.16 | 0.33 | 3.51 | 0.51 | 1.81 | < .001 |
| **Online Sports Betting Frequency** | 0.01 | 0.06 | 0.14 | -0.11 | 0.13 | .892 |
| **Venue Sports Betting Frequency** | 0.10 | 0.07 | 1.43 | -0.04 | 0.23 | .154 |
| **Online Activities Count** | **0.10** | **0.04** | **2.48** | **0.02** | **0.19** | **.013** |
| **Venue Activities Count** | **0.13** | **0.04** | **3.39** | **0.05** | **0.21** | **< .001** |
| **Age** | **-0.01** | **0.00** | **-3.88** | **-0.02** | **-0.01** | **< .001** |
| **Gender (ref. Male)** | 0.11 | 0.09 | 1.31 | -0.06 | 0.28 | .189 |
| **Ethnicity (ref. European)** |  |  |  |  |  |  |
| *Asian* | 0.19 | 0.12 | 1.56 | -0.05 | 0.42 | .118 |
| *Other* | 0.05 | 0.17 | 0.28 | -0.30 | 0.38 | .778 |
| *Indigenous Australian* | 0.11 | 0.23 | 0.49 | -0.37 | 0.53 | .627 |
| **Relationship Status (ref. Not in a romantic relationship)** |  |  |  |  |  |  |
| *Casually dating (i.e., not exclusive)* | 0.07 | 0.18 | 0.39 | -0.29 | 0.40 | .696 |
| *Exclusively dating* | -0.08 | 0.18 | -0.44 | -0.44 | 0.26 | .659 |
| *Engaged* | 0.36 | 0.24 | 1.51 | -0.13 | 0.80 | .131 |
| *Living together* | 0.12 | 0.16 | 0.76 | -0.20 | 0.42 | .450 |
| *Married or defacto* | 0.04 | 0.10 | 0.44 | -0.15 | 0.25 | .662 |
| **Education (ref. Postgraduate qualification)** |  |  |  |  |  |  |
| *University or college degree* | -0.03 | 0.13 | -0.25 | -0.28 | 0.22 | .806 |
| *Trade/technical certificate/diploma* | 0.08 | 0.14 | 0.55 | -0.20 | 0.36 | .582 |
| *Year 12 or equivalent* | 0.02 | 0.15 | 0.10 | -0.27 | 0.31 | .918 |
| *Year 10 or less* | 0.19 | 0.18 | 1.05 | -0.17 | 0.55 | .294 |
| **Work Status (ref. Work full-time)** |  |  |  |  |  |  |
| *Work part-time or casual* | 0.13 | 0.11 | 1.17 | -0.09 | 0.34 | .244 |
| *Full-time student* | 0.08 | 0.19 | 0.41 | -0.30 | 0.44 | .683 |
| *Full-time home duties* | 0.15 | 0.17 | 0.85 | -0.20 | 0.47 | .394 |
| *Unemployed* | -0.15 | 0.14 | -1.06 | -0.42 | 0.12 | .289 |
| **Household Income (ref. Less than $25,000 per year)** |  |  |  |  |  |  |
| *$25,000-$49,999* | -0.25 | 0.16 | -1.51 | -0.56 | 0.08 | .133 |
| *$50,000-$74,999* | -0.18 | 0.17 | -1.05 | -0.51 | 0.16 | .296 |
| *$75,000-$99,999* | **-0.45** | **0.18** | **-2.50** | **-0.81** | **-0.09** | **.013** |
| *$100,000-$124,999* | **-0.59** | **0.20** | **-2.95** | **-0.97** | **-0.20** | **.003** |
| *$125,000-$149,999* | -0.38 | 0.21 | -1.77 | -0.80 | 0.04 | .077 |
| *>$150,000* | -0.18 | 0.20 | -0.90 | -0.56 | 0.21 | .366 |
| *I prefer not to say* | **-0.53** | **0.21** | **-2.56** | **-0.94** | **-0.13** | **.011** |
| **Other Language at Home (ref. Yes)** | -0.02 | 0.13 | -0.15 | -0.28 | 0.24 | .879 |

Note: Adj. *R^2^_v_* = .33. Bolding of model parameters indicates that the coefficient was a significant unique predictor. Levels within categorical predictors were collapsed when there were only a small proportion of participants that endorsed an option (e.g., Ethnicity – Other includes Middle Eastern, African, Latin, Central and South American, and Pacific Islander participants). A single participant selected “Other” as their gender and was omitted from the regression.

*Table S5: Summary of Quasi-Poisson Regression Predicting PGSI Scores for eSports Betting Frequency and Involvement Breadth.*

|  | *B* | SE | *t* | Lower CI | Upper CI | *p* |
| --- | --- | --- | --- | --- | --- | --- |
| **(Intercept)** | 1.31 | 0.32 | 4.11 | 0.68 | 1.93 | < .001 |
| **Online eSports Betting Frequency** | 0.10 | 0.08 | 1.32 | -0.05 | 0.25 | .188 |
| **Venue eSports Betting Frequency** | -0.10 | 0.08 | -1.19 | -0.26 | 0.06 | .236 |
| **Online Activities Count** | **0.09** | **0.04** | **2.10** | **0.01** | **0.17** | **.036** |
| **Venue Activities Count** | **0.17** | **0.04** | **4.34** | **0.09** | **0.24** | **< .001** |
| **Age** | **-0.01** | **0.00** | **-3.80** | **-0.02** | **-0.01** | **< .001** |
| **Gender (ref. Male)** | 0.10 | 0.08 | 1.16 | -0.07 | 0.26 | .245 |
| **Ethnicity (ref. European)** |  |  |  |  |  |  |
| *Asian* | 0.20 | 0.12 | 1.62 | -0.04 | 0.43 | .106 |
| *Other* | 0.06 | 0.17 | 0.35 | -0.29 | 0.39 | .726 |
| *Indigenous Australian* | 0.09 | 0.23 | 0.41 | -0.39 | 0.52 | .681 |
| **Relationship Status (ref. Not in a romantic relationship)** |  |  |  |  |  |  |
| *Casually dating (i.e., not exclusive)* | 0.07 | 0.18 | 0.39 | -0.29 | 0.40 | .699 |
| *Exclusively dating* | -0.05 | 0.18 | -0.30 | -0.41 | 0.28 | .762 |
| *Engaged* | 0.36 | 0.24 | 1.51 | -0.13 | 0.80 | .132 |
| *Living together* | 0.11 | 0.16 | 0.72 | -0.20 | 0.41 | .469 |
| *Married or defacto* | 0.05 | 0.10 | 0.48 | -0.15 | 0.25 | .633 |
| **Education (ref. Postgraduate qualification)** |  |  |  |  |  |  |
| *University or college degree* | -0.03 | 0.13 | -0.20 | -0.27 | 0.23 | .841 |
| *Trade/technical certificate/diploma* | 0.07 | 0.14 | 0.52 | -0.20 | 0.36 | .600 |
| *Year 12 or equivalent* | 0.02 | 0.15 | 0.13 | -0.27 | 0.31 | .894 |
| *Year 10 or less* | 0.20 | 0.18 | 1.12 | -0.15 | 0.56 | .262 |
| **Work Status (ref. Work full-time)** |  |  |  |  |  |  |
| *Work part-time or casual* | 0.11 | 0.11 | 0.98 | -0.11 | 0.32 | .327 |
| *Full-time student* | 0.05 | 0.19 | 0.28 | -0.32 | 0.41 | .777 |
| *Full-time home duties* | 0.13 | 0.17 | 0.73 | -0.22 | 0.45 | .463 |
| *Unemployed* | -0.18 | 0.14 | -1.30 | -0.46 | 0.09 | .195 |
| **Household Income (ref. Less than $25,000 per year)** |  |  |  |  |  |  |
| *$25,000-$49,999* | -0.25 | 0.16 | -1.53 | -0.56 | 0.07 | .127 |
| *$50,000-$74,999* | -0.19 | 0.17 | -1.08 | -0.52 | 0.15 | .280 |
| *$75,000-$99,999* | **-0.47** | **0.18** | **-2.58** | **-0.82** | **-0.11** | **.010** |
| *$100,000-$124,999* | **-0.61** | **0.20** | **-3.06** | **-1.00** | **-0.22** | **.002** |
| *$125,000-$149,999* | -0.40 | 0.21 | -1.91 | -0.82 | 0.01 | .056 |
| *>$150,000* | -0.20 | 0.19 | -1.02 | -0.57 | 0.18 | .309 |
| *I prefer not to say* | **-0.56** | **0.21** | **-2.70** | **-0.97** | **-0.16** | **.007** |
| **Other Language at Home (ref. Yes)** | -0.03 | 0.13 | -0.24 | -0.29 | 0.23 | .814 |

Note: Adj. *R^2^_v_* = .32. Bolding of model parameters indicates that the coefficient was a significant unique predictor. Levels within categorical predictors were collapsed when there were only a small proportion of participants that endorsed an option (e.g., Ethnicity – Other includes Middle Eastern, African, Latin, Central and South American, and Pacific Islander participants). A single participant selected “Other” as their gender and was omitted from the regression.

*Table S6: Summary of Quasi-Poisson Regression Predicting PGSI Scores for Race Wagering Frequency and Involvement Breadth.*

|  | *B* | SE | *t* | Lower CI | Upper CI | *p* |
| --- | --- | --- | --- | --- | --- | --- |
| **(Intercept)** | 1.25 | 0.32 | 3.94 | 0.62 | 1.87 | < .001 |
| **Online Race Wagering Frequency** | 0.04 | 0.06 | 0.67 | -0.08 | 0.16 | .500 |
| **Venue Race Wagering Frequency** | 0.06 | 0.07 | 0.84 | -0.07 | 0.19 | .401 |
| **Online Activities Count** | **0.10** | **0.04** | **2.38** | **0.02** | **0.18** | **.017** |
| **Venue Activities Count** | **0.14** | **0.04** | **3.47** | **0.06** | **0.21** | **< .001** |
| **Age** | **-0.02** | **0.00** | **-4.17** | **-0.02** | **-0.01** | **< .001** |
| **Gender (ref. Male)** | 0.11 | 0.08 | 1.32 | -0.05 | 0.27 | .188 |
| **Ethnicity (ref. European)** |  |  |  |  |  |  |
| *Asian* | 0.19 | 0.12 | 1.57 | -0.05 | 0.42 | .117 |
| *Other* | 0.06 | 0.17 | 0.33 | -0.29 | 0.38 | .738 |
| *Indigenous Australian* | 0.13 | 0.23 | 0.56 | -0.35 | 0.55 | .577 |
| **Relationship Status (ref. Not in a romantic relationship)** |  |  |  |  |  |  |
| *Casually dating (i.e., not exclusive)* | 0.06 | 0.18 | 0.34 | -0.30 | 0.39 | .731 |
| *Exclusively dating* | -0.07 | 0.18 | -0.39 | -0.43 | 0.27 | .693 |
| *Engaged* | 0.33 | 0.24 | 1.38 | -0.16 | 0.77 | .167 |
| *Living together* | 0.13 | 0.16 | 0.81 | -0.19 | 0.43 | .419 |
| *Married or defacto* | 0.04 | 0.10 | 0.42 | -0.16 | 0.24 | .674 |
| **Education (ref. Postgraduate qualification)** |  |  |  |  |  |  |
| *University or college degree* | -0.02 | 0.13 | -0.13 | -0.26 | 0.24 | .897 |
| *Trade/technical certificate/diploma* | 0.07 | 0.14 | 0.52 | -0.20 | 0.36 | .603 |
| *Year 12 or equivalent* | 0.01 | 0.15 | 0.10 | -0.27 | 0.30 | .923 |
| *Year 10 or less* | 0.18 | 0.18 | 1.00 | -0.18 | 0.54 | .318 |
| **Work Status (ref. Work full-time)** |  |  |  |  |  |  |
| *Work part-time or casual* | 0.13 | 0.11 | 1.15 | -0.09 | 0.34 | .249 |
| *Full-time student* | 0.07 | 0.19 | 0.39 | -0.31 | 0.43 | .699 |
| *Full-time home duties* | 0.13 | 0.17 | 0.79 | -0.21 | 0.46 | .431 |
| *Unemployed* | -0.15 | 0.14 | -1.10 | -0.43 | 0.12 | .273 |
| **Household Income (ref. Less than $25,000 per year)** |  |  |  |  |  |  |
| *$25,000-$49,999* | -0.23 | 0.16 | -1.43 | -0.54 | 0.09 | .153 |
| *$50,000-$74,999* | -0.17 | 0.17 | -0.98 | -0.50 | 0.17 | .325 |
| *$75,000-$99,999* | **-0.44** | **0.18** | **-2.43** | **-0.79** | **-0.08** | **.015** |
| *$100,000-$124,999* | **-0.57** | **0.20** | **-2.91** | **-0.96** | **-0.19** | **.004** |
| *$125,000-$149,999* | -0.38 | 0.21 | -1.79 | -0.79 | 0.03 | .073 |
| *>$150,000* | -0.17 | 0.19 | -0.88 | -0.55 | 0.21 | .377 |
| *I prefer not to say* | **-0.53** | **0.21** | **-2.59** | **-0.94** | **-0.13** | **.010** |
| **Other Language at Home (ref. Yes)** | -0.06 | 0.13 | -0.46 | -0.31 | 0.20 | .649 |

Note: Adj. *R^2^_v_* = .32. Bolding of model parameters indicates that the coefficient was a significant unique predictor. Levels within categorical predictors were collapsed when there were only a small proportion of participants that endorsed an option (e.g., Ethnicity – Other includes Middle Eastern, African, Latin, Central and South American, and Pacific Islander participants). A single participant selected “Other” as their gender and was omitted from the regression.

*Table S7: Summary of Quasi-Poisson Regression Predicting PGSI Scores for Poker Frequency and Involvement Breadth.*

|  | *B* | SE | *t* | Lower CI | Upper CI | *p* |
| --- | --- | --- | --- | --- | --- | --- |
| **(Intercept)** | 1.31 | 0.32 | 4.15 | 0.69 | 1.93 | < .001 |
| **Online Poker Frequency** | 0.03 | 0.08 | 0.44 | -0.12 | 0.18 | .658 |
| **Venue Poker Frequency** | -0.02 | 0.08 | -0.25 | -0.18 | 0.14 | .800 |
| **Online Activities Count** | **0.10** | **0.04** | **2.35** | **0.02** | **0.19** | **.019** |
| **Venue Activities Count** | **0.15** | **0.04** | **4.03** | **0.08** | **0.23** | **< .001** |
| **Age** | **-0.01** | **0.00** | **-3.95** | **-0.02** | **-0.01** | **< .001** |
| **Gender (ref. Male)** | 0.10 | 0.08 | 1.14 | -0.07 | 0.26 | .255 |
| **Ethnicity (ref. European)** |  |  |  |  |  |  |
| *Asian* | 0.18 | 0.12 | 1.51 | -0.06 | 0.41 | .130 |
| *Other* | 0.06 | 0.17 | 0.34 | -0.29 | 0.39 | .735 |
| *Indigenous Australian* | 0.11 | 0.23 | 0.48 | -0.37 | 0.54 | .628 |
| **Relationship Status (ref. Not in a romantic relationship)** |  |  |  |  |  |  |
| *Casually dating (i.e., not exclusive)* | 0.06 | 0.18 | 0.33 | -0.30 | 0.39 | .740 |
| *Exclusively dating* | -0.06 | 0.18 | -0.33 | -0.42 | 0.28 | .743 |
| *Engaged* | 0.35 | 0.24 | 1.47 | -0.14 | 0.79 | .142 |
| *Living together* | 0.12 | 0.16 | 0.76 | -0.20 | 0.42 | .450 |
| *Married or defacto* | 0.05 | 0.10 | 0.46 | -0.15 | 0.25 | .648 |
| **Education (ref. Postgraduate qualification)** |  |  |  |  |  |  |
| *University or college degree* | -0.02 | 0.13 | -0.12 | -0.26 | 0.24 | .903 |
| *Trade/technical certificate/diploma* | 0.08 | 0.14 | 0.54 | -0.20 | 0.36 | .587 |
| *Year 12 or equivalent* | 0.02 | 0.15 | 0.13 | -0.27 | 0.31 | .899 |
| *Year 10 or less* | 0.21 | 0.18 | 1.13 | -0.15 | 0.56 | .259 |
| **Work Status (ref. Work full-time)** |  |  |  |  |  |  |
| *Work part-time or casual* | 0.11 | 0.11 | 1.01 | -0.11 | 0.32 | .314 |
| *Full-time student* | 0.06 | 0.19 | 0.29 | -0.33 | 0.42 | .768 |
| *Full-time home duties* | 0.13 | 0.17 | 0.74 | -0.22 | 0.45 | .459 |
| *Unemployed* | -0.17 | 0.14 | -1.24 | -0.45 | 0.10 | .215 |
| **Household Income (ref. Less than $25,000 per year)** |  |  |  |  |  |  |
| *$25,000-$49,999* | -0.23 | 0.16 | -1.45 | -0.55 | 0.09 | .148 |
| *$50,000-$74,999* | -0.17 | 0.17 | -1.01 | -0.51 | 0.17 | .313 |
| *$75,000-$99,999* | **-0.46** | **0.18** | **-2.53** | **-0.81** | **-0.10** | **.012** |
| *$100,000-$124,999* | **-0.59** | **0.20** | **-3.01** | **-0.98** | **-0.21** | **.003** |
| *$125,000-$149,999* | -0.41 | 0.21 | -1.92 | -0.82 | 0.01 | .055 |
| *>$150,000* | -0.20 | 0.19 | -1.02 | -0.58 | 0.18 | .307 |
| *I prefer not to say* | **-0.54** | **0.21** | **-2.61** | **-0.95** | **-0.14** | **.009** |
| **Other Language at Home (ref. Yes)** | -0.05 | 0.13 | -0.37 | -0.30 | 0.22 | .714 |

Note: Adj. *R^2^_v_* = .32. Bolding of model parameters indicates that the coefficient was a significant unique predictor. Levels within categorical predictors were collapsed when there were only a small proportion of participants that endorsed an option (e.g., Ethnicity – Other includes Middle Eastern, African, Latin, Central and South American, and Pacific Islander participants). A single participant selected “Other” as their gender and was omitted from the regression.

*Table S8: Summary of Quasi-Poisson Regression Predicting PGSI Scores for Casino Games Frequency and Involvement Breadth.*

|  | *B* | SE | *t* | Lower CI | Upper CI | *p* |
| --- | --- | --- | --- | --- | --- | --- |
| **(Intercept)** | 1.22 | 0.32 | 3.79 | 0.58 | 1.84 | < .001 |
| **Online Casino Games Frequency** | 0.05 | 0.08 | 0.68 | -0.10 | 0.20 | .498 |
| **Venue Casino Games Frequency** | 0.08 | 0.08 | 0.93 | -0.08 | 0.23 | .352 |
| **Online Activities Count** | **0.09** | **0.04** | **2.06** | **0.00** | **0.18** | **.040** |
| **Venue Activities Count** | **0.13** | **0.04** | **3.37** | **0.06** | **0.21** | **< .001** |
| **Age** | **-0.01** | **0.00** | **-3.91** | **-0.02** | **-0.01** | **< .001** |
| **Gender (ref. Male)** | 0.10 | 0.08 | 1.20 | -0.06 | 0.26 | .231 |
| **Ethnicity (ref. European)** |  |  |  |  |  |  |
| *Asian* | 0.18 | 0.12 | 1.53 | -0.05 | 0.42 | .126 |
| *Other* | 0.06 | 0.17 | 0.34 | -0.29 | 0.39 | .735 |
| *Indigenous Australian* | 0.12 | 0.23 | 0.53 | -0.36 | 0.55 | .599 |
| **Relationship Status (ref. Not in a romantic relationship)** |  |  |  |  |  |  |
| *Casually dating (i.e., not exclusive)* | 0.06 | 0.18 | 0.32 | -0.30 | 0.39 | .753 |
| *Exclusively dating* | -0.05 | 0.18 | -0.26 | -0.41 | 0.29 | .794 |
| *Engaged* | 0.32 | 0.24 | 1.34 | -0.17 | 0.76 | .182 |
| *Living together* | 0.13 | 0.16 | 0.81 | -0.19 | 0.43 | .416 |
| *Married or defacto* | 0.05 | 0.10 | 0.52 | -0.15 | 0.25 | .606 |
| **Education (ref. Postgraduate qualification)** |  |  |  |  |  |  |
| *University or college degree* | -0.01 | 0.13 | -0.07 | -0.26 | 0.24 | .942 |
| *Trade/technical certificate/diploma* | 0.09 | 0.14 | 0.61 | -0.19 | 0.37 | .543 |
| *Year 12 or equivalent* | 0.04 | 0.15 | 0.29 | -0.25 | 0.34 | .771 |
| *Year 10 or less* | 0.23 | 0.18 | 1.27 | -0.13 | 0.59 | .203 |
| **Work Status (ref. Work full-time)** |  |  |  |  |  |  |
| *Work part-time or casual* | 0.11 | 0.11 | 0.97 | -0.11 | 0.32 | .331 |
| *Full-time student* | 0.05 | 0.19 | 0.25 | -0.33 | 0.41 | .804 |
| *Full-time home duties* | 0.14 | 0.17 | 0.81 | -0.21 | 0.46 | .420 |
| *Unemployed* | -0.17 | 0.14 | -1.24 | -0.45 | 0.10 | .215 |
| **Household Income (ref. Less than $25,000 per year)** |  |  |  |  |  |  |
| *$25,000-$49,999* | -0.24 | 0.16 | -1.48 | -0.55 | 0.08 | .138 |
| *$50,000-$74,999* | -0.18 | 0.17 | -1.07 | -0.52 | 0.16 | .287 |
| *$75,000-$99,999* | **-0.46** | **0.18** | **-2.55** | **-0.82** | **-0.10** | **.011** |
| *$100,000-$124,999* | **-0.60** | **0.20** | **-3.03** | **-0.99** | **-0.21** | **.002** |
| *$125,000-$149,999* | -0.40 | 0.21 | -1.89 | -0.82 | 0.01 | .059 |
| *>$150,000* | -0.21 | 0.19 | -1.08 | -0.59 | 0.17 | .281 |
| *I prefer not to say* | **-0.56** | **0.21** | **-2.72** | **-0.97** | **-0.16** | **.007** |
| **Other Language at Home (ref. Yes)** | -0.04 | 0.13 | -0.32 | -0.30 | 0.22 | .750 |

Note: Adj. *R^2^_v_* = .33. Bolding of model parameters indicates that the coefficient was a significant unique predictor. Levels within categorical predictors were collapsed when there were only a small proportion of participants that endorsed an option (e.g., Ethnicity – Other includes Middle Eastern, African, Latin, Central and South American, and Pacific Islander participants). A single participant selected “Other” as their gender and was omitted from the regression.

*Table S9: Summary of Quasi-Poisson Regression Predicting Kessler 6 Scores for EGM Frequency and Involvement Breadth.*

|  | *B* | SE | *t* | Lower CI | Upper CI | *p* |
| --- | --- | --- | --- | --- | --- | --- |
| **(Intercept)** | 1.93 | 0.27 | 7.20 | 1.40 | 2.45 | < .001 |
| **Online EGM Frequency** | 0.09 | 0.06 | 1.49 | -0.03 | 0.20 | .137 |
| **Venue EGM Frequency** | 0.10 | 0.06 | 1.71 | -0.01 | 0.20 | .087 |
| **Online Activities Count** | 0.02 | 0.04 | 0.65 | -0.05 | 0.09 | .517 |
| **Venue Activities Count** | 0.05 | 0.03 | 1.65 | -0.01 | 0.11 | .099 |
| **Age** | **-0.01** | **0.00** | **-5.08** | **-0.02** | **-0.01** | **< .001** |
| **Gender (ref. Male)** | 0.06 | 0.07 | 0.87 | -0.08 | 0.20 | .387 |
| **Ethnicity (ref. European)** |  |  |  |  |  |  |
| *Asian* | 0.00 | 0.11 | 0.01 | -0.22 | 0.21 | .995 |
| *Other* | 0.06 | 0.15 | 0.44 | -0.23 | 0.34 | .662 |
| *Indigenous Australian* | 0.19 | 0.19 | 0.98 | -0.21 | 0.54 | .329 |
| **Relationship Status (ref. Not in a romantic relationship)** |  |  |  |  |  |  |
| *Casually dating (i.e., not exclusive)* | -0.01 | 0.15 | -0.07 | -0.32 | 0.28 | .944 |
| *Exclusively dating* | -0.05 | 0.15 | -0.35 | -0.35 | 0.23 | .727 |
| *Engaged* | -0.01 | 0.23 | -0.04 | -0.48 | 0.41 | .966 |
| *Living together* | -0.17 | 0.14 | -1.23 | -0.44 | 0.09 | .218 |
| *Married or defacto* | -0.16 | 0.08 | -1.91 | -0.32 | 0.00 | .057 |
| **Education (ref. Postgraduate qualification)** |  |  |  |  |  |  |
| *University or college degree* | 0.16 | 0.12 | 1.39 | -0.06 | 0.39 | .165 |
| *Trade/technical certificate/diploma* | 0.08 | 0.13 | 0.64 | -0.16 | 0.33 | .519 |
| *Year 12 or equivalent* | 0.15 | 0.13 | 1.16 | -0.10 | 0.41 | .246 |
| *Year 10 or less* | 0.14 | 0.15 | 0.95 | -0.15 | 0.44 | .340 |
| **Work Status (ref. Work full-time)** |  |  |  |  |  |  |
| *Work part-time or casual* | -0.02 | 0.09 | -0.22 | -0.20 | 0.16 | .828 |
| *Full-time student* | -0.29 | 0.17 | -1.69 | -0.64 | 0.04 | .092 |
| *Full-time home duties* | -0.18 | 0.15 | -1.20 | -0.50 | 0.11 | .230 |
| *Unemployed* | -0.01 | 0.11 | -0.08 | -0.22 | 0.20 | .936 |
| **Household Income (ref. Less than $25,000 per year)** |  |  |  |  |  |  |
| *$25,000-$49,999* | -0.15 | 0.14 | -1.08 | -0.41 | 0.12 | .281 |
| *$50,000-$74,999* | 0.11 | 0.14 | 0.76 | -0.17 | 0.39 | .448 |
| *$75,000-$99,999* | -0.11 | 0.15 | -0.70 | -0.40 | 0.19 | .486 |
| *$100,000-$124,999* | -0.25 | 0.17 | -1.48 | -0.57 | 0.08 | .138 |
| *$125,000-$149,999* | -0.07 | 0.18 | -0.42 | -0.42 | 0.27 | .674 |
| *>$150,000* | -0.12 | 0.17 | -0.68 | -0.45 | 0.22 | .500 |
| *I prefer not to say* | -0.24 | 0.17 | -1.44 | -0.57 | 0.09 | .151 |
| **Other Language at Home (ref. Yes)** | -0.02 | 0.12 | -0.18 | -0.25 | 0.22 | .861 |

Note: Adj. *R^2^_v_* = .15. Bolding of model parameters indicates that the coefficient was a significant unique predictor. Levels within categorical predictors were collapsed when there were only a small proportion of participants that endorsed an option (e.g., Ethnicity – Other includes Middle Eastern, African, Latin, Central and South American, and Pacific Islander participants). A single participant selected “Other” as their gender and was omitted from the regression.

*Table S10: Summary of Quasi-Poisson Regression Predicting Kessler 6 Scores for Lottery Frequency and Involvement Breadth.*

|  | *B* | SE | *t* | Lower CI | Upper CI | *p* |
| --- | --- | --- | --- | --- | --- | --- |
| **(Intercept)** | 1.96 | 0.27 | 7.20 | 1.42 | 2.49 | < .001 |
| **Online Lottery Frequency** | 0.08 | 0.05 | 1.65 | -0.01 | 0.17 | .099 |
| **Venue Lottery Frequency** | 0.00 | 0.05 | 0.01 | -0.10 | 0.10 | .991 |
| **Online Activities Count** | 0.03 | 0.04 | 0.99 | -0.03 | 0.10 | .323 |
| **Venue Activities Count** | **0.08** | **0.03** | **2.23** | **0.01** | **0.14** | **.026** |
| **Age** | **-0.02** | **0.00** | **-5.11** | **-0.02** | **-0.01** | **< .001** |
| **Gender (ref. Male)** | 0.06 | 0.07 | 0.79 | -0.08 | 0.20 | .428 |
| **Ethnicity (ref. European)** |  |  |  |  |  |  |
| *Asian* | -0.01 | 0.11 | -0.05 | -0.22 | 0.21 | .962 |
| *Other* | 0.04 | 0.15 | 0.26 | -0.26 | 0.32 | .792 |
| *Indigenous Australian* | 0.17 | 0.19 | 0.90 | -0.22 | 0.53 | .368 |
| **Relationship Status (ref. Not in a romantic relationship)** |  |  |  |  |  |  |
| *Casually dating (i.e., not exclusive)* | 0.03 | 0.15 | 0.18 | -0.28 | 0.32 | .857 |
| *Exclusively dating* | -0.07 | 0.15 | -0.47 | -0.37 | 0.21 | .640 |
| *Engaged* | 0.05 | 0.23 | 0.24 | -0.42 | 0.48 | .812 |
| *Living together* | -0.17 | 0.14 | -1.25 | -0.44 | 0.09 | .210 |
| *Married or defacto* | **-0.17** | **0.08** | **-2.05** | **-0.33** | **-0.01** | **.041** |
| **Education (ref. Postgraduate qualification)** |  |  |  |  |  |  |
| *University or college degree* | 0.17 | 0.12 | 1.44 | -0.06 | 0.40 | .151 |
| *Trade/technical certificate/diploma* | 0.10 | 0.13 | 0.80 | -0.14 | 0.35 | .425 |
| *Year 12 or equivalent* | 0.19 | 0.13 | 1.47 | -0.06 | 0.45 | .142 |
| *Year 10 or less* | 0.18 | 0.15 | 1.20 | -0.12 | 0.48 | .230 |
| **Work Status (ref. Work full-time)** |  |  |  |  |  |  |
| *Work part-time or casual* | -0.01 | 0.09 | -0.08 | -0.19 | 0.17 | .940 |
| *Full-time student* | -0.26 | 0.17 | -1.52 | -0.61 | 0.07 | .128 |
| *Full-time home duties* | -0.16 | 0.15 | -1.03 | -0.47 | 0.14 | .304 |
| *Unemployed* | 0.00 | 0.11 | 0.01 | -0.21 | 0.21 | .991 |
| **Household Income (ref. Less than $25,000 per year)** |  |  |  |  |  |  |
| *$25,000-$49,999* | -0.15 | 0.14 | -1.08 | -0.41 | 0.13 | .282 |
| *$50,000-$74,999* | 0.12 | 0.14 | 0.82 | -0.16 | 0.40 | .415 |
| *$75,000-$99,999* | -0.09 | 0.15 | -0.59 | -0.39 | 0.21 | .558 |
| *$100,000-$124,999* | -0.26 | 0.17 | -1.56 | -0.59 | 0.07 | .119 |
| *$125,000-$149,999* | -0.05 | 0.18 | -0.31 | -0.40 | 0.29 | .757 |
| *>$150,000* | -0.06 | 0.17 | -0.34 | -0.40 | 0.28 | .732 |
| *I prefer not to say* | -0.23 | 0.17 | -1.40 | -0.57 | 0.09 | .163 |
| **Other Language at Home (ref. Yes)** | -0.02 | 0.12 | -0.21 | -0.26 | 0.21 | .837 |

Note: Adj. *R^2^_v_* = .15. Bolding of model parameters indicates that the coefficient was a significant unique predictor. Levels within categorical predictors were collapsed when there were only a small proportion of participants that endorsed an option (e.g., Ethnicity – Other includes Middle Eastern, African, Latin, Central and South American, and Pacific Islander participants). A single participant selected “Other” as their gender and was omitted from the regression.

*Table S11: Summary of Quasi-Poisson Regression Predicting Kessler 6 Scores for Sports Betting Frequency and Involvement Breadth.*

|  | *B* | SE | *t* | Lower CI | Upper CI | *p* |
| --- | --- | --- | --- | --- | --- | --- |
| **(Intercept)** | 2.05 | 0.28 | 7.39 | 1.51 | 2.60 | < .001 |
| **Online Sports Betting Frequency** | -0.09 | 0.05 | -1.62 | -0.19 | 0.02 | .106 |
| **Venue Sports Betting Frequency** | 0.10 | 0.06 | 1.54 | -0.03 | 0.22 | .124 |
| **Online Activities Count** | **0.07** | **0.04** | **2.01** | **0.00** | **0.14** | **.045** |
| **Venue Activities Count** | 0.05 | 0.03 | 1.55 | -0.01 | 0.11 | .122 |
| **Age** | **-0.01** | **0.00** | **-5.03** | **-0.02** | **-0.01** | **< .001** |
| **Gender (ref. Male)** | 0.06 | 0.07 | 0.85 | -0.08 | 0.20 | .397 |
| **Ethnicity (ref. European)** |  |  |  |  |  |  |
| *Asian* | -0.02 | 0.11 | -0.18 | -0.24 | 0.19 | .853 |
| *Other* | 0.05 | 0.15 | 0.34 | -0.25 | 0.33 | .736 |
| *Indigenous Australian* | 0.20 | 0.19 | 1.06 | -0.19 | 0.56 | .289 |
| **Relationship Status (ref. Not in a romantic relationship)** |  |  |  |  |  |  |
| *Casually dating (i.e., not exclusive)* | 0.03 | 0.15 | 0.18 | -0.28 | 0.32 | .861 |
| *Exclusively dating* | -0.07 | 0.15 | -0.43 | -0.37 | 0.22 | .664 |
| *Engaged* | 0.06 | 0.23 | 0.26 | -0.41 | 0.48 | .797 |
| *Living together* | -0.16 | 0.14 | -1.19 | -0.44 | 0.10 | .235 |
| *Married or defacto* | **-0.17** | **0.08** | **-2.03** | **-0.33** | **-0.01** | **.042** |
| **Education (ref. Postgraduate qualification)** |  |  |  |  |  |  |
| *University or college degree* | 0.17 | 0.12 | 1.48 | -0.05 | 0.41 | .138 |
| *Trade/technical certificate/diploma* | 0.12 | 0.13 | 0.92 | -0.13 | 0.37 | .358 |
| *Year 12 or equivalent* | 0.19 | 0.13 | 1.48 | -0.06 | 0.45 | .140 |
| *Year 10 or less* | 0.19 | 0.15 | 1.22 | -0.11 | 0.49 | .224 |
| **Work Status (ref. Work full-time)** |  |  |  |  |  |  |
| *Work part-time or casual* | -0.02 | 0.09 | -0.16 | -0.20 | 0.17 | .870 |
| *Full-time student* | -0.29 | 0.17 | -1.66 | -0.64 | 0.04 | .096 |
| *Full-time home duties* | -0.15 | 0.15 | -0.98 | -0.46 | 0.14 | .329 |
| *Unemployed* | -0.00 | 0.11 | -0.03 | -0.21 | 0.20 | .978 |
| **Household Income (ref. Less than $25,000 per year)** |  |  |  |  |  |  |
| *$25,000-$49,999* | -0.14 | 0.14 | -0.98 | -0.40 | 0.14 | .328 |
| *$50,000-$74,999* | 0.13 | 0.14 | 0.93 | -0.15 | 0.42 | .354 |
| *$75,000-$99,999* | -0.07 | 0.15 | -0.49 | -0.37 | 0.23 | .623 |
| *$100,000-$124,999* | -0.23 | 0.17 | -1.40 | -0.56 | 0.09 | .162 |
| *$125,000-$149,999* | -0.03 | 0.18 | -0.14 | -0.38 | 0.32 | .886 |
| *>$150,000* | -0.04 | 0.17 | -0.24 | -0.38 | 0.30 | .811 |
| *I prefer not to say* | -0.22 | 0.17 | -1.30 | -0.55 | 0.11 | .193 |
| **Other Language at Home (ref. Yes)** | -0.01 | 0.12 | -0.10 | -0.25 | 0.23 | .921 |

Note: Adj. *R^2^_v_* = .15. Bolding of model parameters indicates that the coefficient was a significant unique predictor. Levels within categorical predictors were collapsed when there were only a small proportion of participants that endorsed an option (e.g., Ethnicity – Other includes Middle Eastern, African, Latin, Central and South American, and Pacific Islander participants). A single participant selected “Other” as their gender and was omitted from the regression.

*Table S12: Summary of Quasi-Poisson Regression Predicting Kessler 6 Scores for eSports Betting Frequency and Involvement Breadth.*

|  | *B* | SE | *t* | Lower CI | Upper CI | *p* |
| --- | --- | --- | --- | --- | --- | --- |
| **(Intercept)** | 2.11 | 0.27 | 7.82 | 1.58 | 2.64 | < .001 |
| **Online eSports Betting Frequency** | 0.01 | 0.07 | 0.11 | -0.14 | 0.15 | .910 |
| **Venue eSports Betting Frequency** | -0.10 | 0.08 | -1.19 | -0.25 | 0.06 | .235 |
| **Online Activities Count** | 0.05 | 0.04 | 1.52 | -0.02 | 0.12 | .130 |
| **Venue Activities Count** | **0.09** | **0.03** | **2.94** | **0.03** | **0.15** | **.003** |
| **Age** | **-0.01** | **0.00** | **-4.93** | **-0.02** | **-0.01** | **< .001** |
| **Gender (ref. Male)** | 0.07 | 0.07 | 1.03 | -0.07 | 0.21 | .303 |
| **Ethnicity (ref. European)** |  |  |  |  |  |  |
| *Asian* | 0.01 | 0.11 | 0.08 | -0.21 | 0.22 | .933 |
| *Other* | 0.07 | 0.15 | 0.45 | -0.23 | 0.34 | .655 |
| *Indigenous Australian* | 0.20 | 0.19 | 1.05 | -0.19 | 0.56 | .295 |
| **Relationship Status (ref. Not in a romantic relationship)** |  |  |  |  |  |  |
| *Casually dating (i.e., not exclusive)* | 0.01 | 0.15 | 0.08 | -0.30 | 0.30 | .936 |
| *Exclusively dating* | -0.05 | 0.15 | -0.33 | -0.35 | 0.24 | .744 |
| *Engaged* | 0.05 | 0.23 | 0.23 | -0.42 | 0.48 | .820 |
| *Living together* | -0.18 | 0.14 | -1.29 | -0.45 | 0.09 | .199 |
| *Married or defacto* | -0.18 | 0.08 | -2.13 | -0.34 | -0.01 | .034 |
| **Education (ref. Postgraduate qualification)** |  |  |  |  |  |  |
| *University or college degree* | 0.17 | 0.12 | 1.46 | -0.05 | 0.40 | .144 |
| *Trade/technical certificate/diploma* | 0.10 | 0.13 | 0.78 | -0.15 | 0.35 | .438 |
| *Year 12 or equivalent* | 0.17 | 0.13 | 1.31 | -0.08 | 0.43 | .189 |
| *Year 10 or less* | 0.17 | 0.15 | 1.09 | -0.13 | 0.46 | .278 |
| **Work Status (ref. Work full-time)** |  |  |  |  |  |  |
| *Work part-time or casual* | -0.01 | 0.09 | -0.08 | -0.19 | 0.17 | .935 |
| *Full-time student* | -0.27 | 0.17 | -1.57 | -0.62 | 0.06 | .116 |
| *Full-time home duties* | -0.15 | 0.15 | -0.99 | -0.46 | 0.14 | .321 |
| *Unemployed* | 0.01 | 0.11 | 0.09 | -0.20 | 0.22 | .926 |
| **Household Income (ref. Less than $25,000 per year)** |  |  |  |  |  |  |
| *$25,000-$49,999* | -0.16 | 0.14 | -1.15 | -0.42 | 0.12 | .251 |
| *$50,000-$74,999* | 0.13 | 0.14 | 0.88 | -0.15 | 0.41 | .381 |
| *$75,000-$99,999* | -0.10 | 0.15 | -0.65 | -0.40 | 0.20 | .514 |
| *$100,000-$124,999* | -0.27 | 0.17 | -1.64 | -0.60 | 0.05 | .102 |
| *$125,000-$149,999* | -0.07 | 0.18 | -0.38 | -0.42 | 0.28 | .701 |
| *>$150,000* | -0.08 | 0.17 | -0.44 | -0.42 | 0.26 | .657 |
| *I prefer not to say* | -0.24 | 0.17 | -1.44 | -0.57 | 0.09 | .151 |
| **Other Language at Home (ref. Yes)** | -0.01 | 0.12 | -0.05 | -0.24 | 0.23 | .960 |

Note: Adj. *R^2^_v_* = .15. Bolding of model parameters indicates that the coefficient was a significant unique predictor. Levels within categorical predictors were collapsed when there were only a small proportion of participants that endorsed an option (e.g., Ethnicity – Other includes Middle Eastern, African, Latin, Central and South American, and Pacific Islander participants). A single participant selected “Other” as their gender and was omitted from the regression.

*Table S13: Summary of Quasi-Poisson Regression Predicting Kessler 6 Scores for Race Wagering Frequency and Involvement Breadth.*

|  | *B* | SE | *t* | Lower CI | Upper CI | *p* |
| --- | --- | --- | --- | --- | --- | --- |
| **(Intercept)** | 2.13 | 0.27 | 7.95 | 1.60 | 2.65 | < .001 |
| **Online Race Wagering Frequency** | -0.08 | 0.05 | -1.62 | -0.18 | 0.02 | .105 |
| **Venue Race Wagering Frequency** | -0.04 | 0.06 | -0.72 | -0.16 | 0.07 | .472 |
| **Online Activities Count** | **0.07** | **0.03** | **2.08** | **0.00** | **0.14** | **.038** |
| **Venue Activities Count** | **0.09** | **0.03** | **2.70** | **0.02** | **0.15** | **.007** |
| **Age** | **-0.01** | **0.00** | **-4.48** | **-0.02** | **-0.01** | **< .001** |
| **Gender (ref. Male)** | 0.05 | 0.07 | 0.75 | -0.09 | 0.19 | .454 |
| **Ethnicity (ref. European)** |  |  |  |  |  |  |
| *Asian* | -0.02 | 0.11 | -0.16 | -0.24 | 0.20 | .872 |
| *Other* | 0.05 | 0.15 | 0.34 | -0.25 | 0.33 | .735 |
| *Indigenous Australian* | 0.18 | 0.19 | 0.96 | -0.21 | 0.54 | .338 |
| **Relationship Status (ref. Not in a romantic relationship)** |  |  |  |  |  |  |
| *Casually dating (i.e., not exclusive)* | 0.01 | 0.15 | 0.07 | -0.30 | 0.30 | .942 |
| *Exclusively dating* | -0.06 | 0.15 | -0.38 | -0.36 | 0.23 | .702 |
| *Engaged* | 0.05 | 0.23 | 0.20 | -0.43 | 0.47 | .839 |
| *Living together* | -0.18 | 0.14 | -1.33 | -0.46 | 0.08 | .182 |
| *Married or defacto* | **-0.16** | **0.08** | **-1.99** | **-0.32** | **-0.00** | **.047** |
| **Education (ref. Postgraduate qualification)** |  |  |  |  |  |  |
| *University or college degree* | 0.17 | 0.12 | 1.46 | -0.05 | 0.40 | .145 |
| *Trade/technical certificate/diploma* | 0.11 | 0.13 | 0.84 | -0.14 | 0.36 | .400 |
| *Year 12 or equivalent* | 0.19 | 0.13 | 1.46 | -0.06 | 0.45 | .144 |
| *Year 10 or less* | 0.21 | 0.15 | 1.38 | -0.09 | 0.51 | .168 |
| **Work Status (ref. Work full-time)** |  |  |  |  |  |  |
| *Work part-time or casual* | -0.03 | 0.09 | -0.34 | -0.22 | 0.15 | .731 |
| *Full-time student* | -0.30 | 0.17 | -1.72 | -0.65 | 0.03 | .086 |
| *Full-time home duties* | -0.16 | 0.15 | -1.01 | -0.47 | 0.14 | .313 |
| *Unemployed* | -0.01 | 0.11 | -0.12 | -0.22 | 0.19 | .901 |
| **Household Income (ref. Less than $25,000 per year)** |  |  |  |  |  |  |
| *$25,000-$49,999* | -0.15 | 0.14 | -1.11 | -0.42 | 0.12 | .266 |
| *$50,000-$74,999* | 0.12 | 0.14 | 0.87 | -0.15 | 0.41 | .385 |
| *$75,000-$99,999* | -0.11 | 0.15 | -0.72 | -0.41 | 0.19 | .474 |
| *$100,000-$124,999* | -0.28 | 0.17 | -1.66 | -0.60 | 0.05 | .097 |
| *$125,000-$149,999* | -0.08 | 0.18 | -0.48 | -0.43 | 0.26 | .634 |
| *>$150,000* | -0.09 | 0.17 | -0.54 | -0.43 | 0.25 | .590 |
| *I prefer not to say* | -0.24 | 0.17 | -1.42 | -0.57 | 0.09 | .155 |
| **Other Language at Home (ref. Yes)** | -0.00 | 0.12 | -0.03 | -0.24 | 0.24 | .975 |

Note: Adj. *R^2^_v_* = .15. Bolding of model parameters indicates that the coefficient was a significant unique predictor. Levels within categorical predictors were collapsed when there were only a small proportion of participants that endorsed an option (e.g., Ethnicity – Other includes Middle Eastern, African, Latin, Central and South American, and Pacific Islander participants). A single participant selected “Other” as their gender and was omitted from the regression.

*Table S14: Summary of Quasi-Poisson Regression Predicting Kessler 6 Scores for Poker Frequency and Involvement Breadth.*

|  | *B* | SE | *t* | Lower CI | Upper CI | *p* |
| --- | --- | --- | --- | --- | --- | --- |
| **(Intercept)** | 2.04 | 0.27 | 7.62 | 1.51 | 2.56 | < .001 |
| **Online Poker Frequency** | 0.10 | 0.07 | 1.50 | -0.03 | 0.24 | .134 |
| **Venue Poker Frequency** | -0.10 | 0.08 | -1.31 | -0.25 | 0.05 | .191 |
| **Online Activities Count** | 0.03 | 0.04 | 0.85 | -0.04 | 0.10 | .396 |
| **Venue Activities Count** | **0.09** | **0.03** | **2.91** | **0.03** | **0.15** | **.004** |
| **Age** | **-0.01** | **0.00** | **-4.89** | **-0.02** | **-0.01** | **< .001** |
| **Gender (ref. Male)** | 0.08 | 0.07 | 1.16 | -0.06 | 0.22 | .247 |
| **Ethnicity (ref. European)** |  |  |  |  |  |  |
| *Asian* | -0.00 | 0.11 | -0.02 | -0.22 | 0.21 | .988 |
| *Other* | 0.06 | 0.15 | 0.40 | -0.24 | 0.33 | .692 |
| *Indigenous Australian* | 0.19 | 0.19 | 0.99 | -0.20 | 0.54 | .322 |
| **Relationship Status (ref. Not in a romantic relationship)** |  |  |  |  |  |  |
| *Casually dating (i.e., not exclusive)* | 0.01 | 0.15 | 0.05 | -0.30 | 0.30 | .959 |
| *Exclusively dating* | -0.05 | 0.15 | -0.36 | -0.36 | 0.23 | .722 |
| *Engaged* | 0.03 | 0.23 | 0.13 | -0.44 | 0.45 | .894 |
| *Living together* | -0.18 | 0.14 | -1.29 | -0.45 | 0.09 | .196 |
| *Married or defacto* | -0.17 | 0.08 | -2.11 | -0.33 | -0.01 | .035 |
| **Education (ref. Postgraduate qualification)** |  |  |  |  |  |  |
| *University or college degree* | 0.18 | 0.12 | 1.51 | -0.05 | 0.41 | .132 |
| *Trade/technical certificate/diploma* | 0.11 | 0.13 | 0.84 | -0.14 | 0.36 | .403 |
| *Year 12 or equivalent* | 0.18 | 0.13 | 1.35 | -0.08 | 0.43 | .176 |
| *Year 10 or less* | 0.18 | 0.15 | 1.16 | -0.12 | 0.48 | .245 |
| **Work Status (ref. Work full-time)** |  |  |  |  |  |  |
| *Work part-time or casual* | -0.01 | 0.09 | -0.11 | -0.19 | 0.17 | .914 |
| *Full-time student* | -0.26 | 0.17 | -1.50 | -0.61 | 0.07 | .135 |
| *Full-time home duties* | -0.15 | 0.15 | -0.95 | -0.46 | 0.15 | .341 |
| *Unemployed* | 0.00 | 0.11 | 0.02 | -0.21 | 0.21 | .985 |
| **Household Income (ref. Less than $25,000 per year)** |  |  |  |  |  |  |
| *$25,000-$49,999* | -0.15 | 0.14 | -1.11 | -0.42 | 0.12 | .265 |
| *$50,000-$74,999* | 0.12 | 0.14 | 0.86 | -0.16 | 0.41 | .389 |
| *$75,000-$99,999* | -0.09 | 0.15 | -0.62 | -0.39 | 0.21 | .535 |
| *$100,000-$124,999* | -0.26 | 0.17 | -1.53 | -0.58 | 0.07 | .125 |
| *$125,000-$149,999* | -0.07 | 0.18 | -0.38 | -0.42 | 0.28 | .707 |
| *>$150,000* | -0.09 | 0.17 | -0.50 | -0.42 | 0.25 | .620 |
| *I prefer not to say* | -0.23 | 0.17 | -1.37 | -0.56 | 0.10 | .170 |
| **Other Language at Home (ref. Yes)** | -0.00 | 0.12 | -0.03 | -0.24 | 0.24 | .977 |

Note: Adj. *R^2^_v_* = .15. Bolding of model parameters indicates that the coefficient was a significant unique predictor. Levels within categorical predictors were collapsed when there were only a small proportion of participants that endorsed an option (e.g., Ethnicity – Other includes Middle Eastern, African, Latin, Central and South American, and Pacific Islander participants). A single participant selected “Other” as their gender and was omitted from the regression.

*Table S15: Summary of Quasi-Poisson Regression Predicting Kessler 6 Scores for Casino Games Frequency and Involvement Breadth.*

|  | *B* | SE | *t* | Lower CI | Upper CI | *p* |
| --- | --- | --- | --- | --- | --- | --- |
| **(Intercept)** | 1.97 | 0.27 | 7.26 | 1.43 | 2.50 | < .001 |
| **Online Casino Games Frequency** | 0.03 | 0.07 | 0.41 | -0.11 | 0.16 | .681 |
| **Venue Casino Games Frequency** | 0.07 | 0.07 | 1.00 | -0.07 | 0.22 | .317 |
| **Online Activities Count** | 0.04 | 0.04 | 1.05 | -0.03 | 0.11 | .294 |
| **Venue Activities Count** | 0.06 | 0.03 | 1.84 | -0.00 | 0.12 | .066 |
| **Age** | **-0.01** | **0.00** | **-4.91** | **-0.02** | **-0.01** | **< .001** |
| **Gender (ref. Male)** | 0.08 | 0.07 | 1.19 | -0.05 | 0.22 | .236 |
| **Ethnicity (ref. European)** |  |  |  |  |  |  |
| *Asian* | -0.01 | 0.11 | -0.07 | -0.23 | 0.21 | .944 |
| *Other* | 0.05 | 0.15 | 0.37 | -0.24 | 0.33 | .708 |
| *Indigenous Australian* | 0.20 | 0.19 | 1.05 | -0.19 | 0.55 | .296 |
| **Relationship Status (ref. Not in a romantic relationship)** |  |  |  |  |  |  |
| *Casually dating (i.e., not exclusive)* | 0.01 | 0.15 | 0.04 | -0.30 | 0.30 | .967 |
| *Exclusively dating* | -0.05 | 0.15 | -0.32 | -0.35 | 0.24 | .746 |
| *Engaged* | 0.02 | 0.23 | 0.08 | -0.46 | 0.44 | .938 |
| *Living together* | -0.16 | 0.14 | -1.21 | -0.44 | 0.10 | .227 |
| *Married or defacto* | **-0.16** | **0.08** | **-1.99** | **-0.32** | **-0.00** | **.047** |
| **Education (ref. Postgraduate qualification)** |  |  |  |  |  |  |
| *University or college degree* | 0.18 | 0.12 | 1.51 | -0.05 | 0.41 | .132 |
| *Trade/technical certificate/diploma* | 0.11 | 0.13 | 0.86 | -0.14 | 0.36 | .392 |
| *Year 12 or equivalent* | 0.19 | 0.13 | 1.47 | -0.06 | 0.45 | .141 |
| *Year 10 or less* | 0.20 | 0.15 | 1.31 | -0.10 | 0.50 | .192 |
| **Work Status (ref. Work full-time)** |  |  |  |  |  |  |
| *Work part-time or casual* | -0.01 | 0.09 | -0.15 | -0.20 | 0.17 | .881 |
| *Full-time student* | -0.28 | 0.17 | -1.62 | -0.63 | 0.05 | .106 |
| *Full-time home duties* | -0.14 | 0.15 | -0.91 | -0.45 | 0.15 | .364 |
| *Unemployed* | 0.00 | 0.11 | 0.04 | -0.20 | 0.21 | .971 |
| **Household Income (ref. Less than $25,000 per year)** |  |  |  |  |  |  |
| *$25,000-$49,999* | -0.16 | 0.14 | -1.13 | -0.42 | 0.12 | .259 |
| *$50,000-$74,999* | 0.12 | 0.14 | 0.85 | -0.16 | 0.41 | .398 |
| *$75,000-$99,999* | -0.10 | 0.15 | -0.63 | -0.39 | 0.21 | .531 |
| *$100,000-$124,999* | -0.26 | 0.17 | -1.57 | -0.59 | 0.07 | .117 |
| *$125,000-$149,999* | -0.06 | 0.18 | -0.35 | -0.41 | 0.29 | .726 |
| *>$150,000* | -0.09 | 0.17 | -0.50 | -0.43 | 0.25 | .614 |
| *I prefer not to say* | -0.25 | 0.17 | -1.47 | -0.58 | 0.08 | .141 |
| **Other Language at Home (ref. Yes)** | -0.01 | 0.12 | -0.11 | -0.25 | 0.23 | .911 |

Note: Adj. *R^2^_v_* = .15. Bolding of model parameters indicates that the coefficient was a significant unique predictor. Levels within categorical predictors were collapsed when there were only a small proportion of participants that endorsed an option (e.g., Ethnicity – Other includes Middle Eastern, African, Latin, Central and South American, and Pacific Islander participants). A single participant selected “Other” as their gender and was omitted from the regression.
